# Direct neuronal reprogramming for neurological diseases: applications, translational Challenges, and future directions

**DOI:** 10.3389/fnins.2026.1889859

**Published:** 2026-08-07

**Authors:** Yu Chen, Zhe Zheng, Peiqi Zhao, Jiajie Zhu, Hangzhe Sun, Haoliang Zhu, Yaying Zeng, Bowen Shang, Anke Zhang, Yuanbo Pan, Kankai Wang

**Affiliations:** 1 School of Medicine, The Second Affiliated Hospital, Zhejiang University, Hangzhou, China; 2 National Clinical Research Center for Ocular Diseases, Eye Hospital, Wenzhou Medical University, Wenzhou, China; 3 Department of Neurosurgery, The Second Affiliated Hospital, School of Medicine, Zhejiang University, Hangzhou, China; 4 Key Laboratory of Precise Treatment and Clinical Translational Research of Neurological Diseases, Hangzhou, China; 5 Zhejiang Provincial Key Laboratory of Aging and Neurological Disorder Research, The First Affiliated Hospital of Wenzhou Medical University, Wenzhou, China; 6 The First Clinical Medical College, Wenzhou Medical University, Wenzhou, China; 7 Department of Psychiatry, The First Affiliated Hospital of Zhejiang Chinese Medical University (Zhejiang Provincial Hospital of Traditional Chinese Medicine), Hangzhou, China; 8 Department of Neurosurgery, The First Affiliated Hospital of Bengbu Medical University, Bengbu, China

**Keywords:** direct neuronal reprogramming, induced pluripotent stem cells, neurological diseases, neuroregeneration, transcription factor

## Abstract

Neurological diseases, often caused by irreversible loss of terminally differentiated neurons, present considerable challenges to treatment due to the limited regenerative capacity of these neurons. Although induced pluripotent stem cells hold promise for neuronal regeneration, their clinical application is constrained by risks, including tumorigenicity, incomplete neuronal maturation, and immune rejection. Recent advancements in direct neuronal reprogramming, which bypasses the intermediate pluripotent stage by directly converting non-neuronal cells into functional neurons, offer a compelling alternative for *in situ* neuronal replacement in neurodegenerative diseases. Key transcription factors, such as NeuroD1, Ascl1, Sox2, as well as CRISPR activation (CRISPRa) of NGN2 and ISL1, have been explored to convert glial cells into neurons. However, several challenges remain. This review discusses the current applications of direct neuronal reprogramming technology in several neurological diseases. We further highlight the potential contamination issues in adeno-associated virus (AAV) delivery systems and propose a code of conduct to avoid artifacts and pitfalls. Finally, we point out future directions for expanding direct reprogramming targets, integrating organoid-based disease modeling, and advancing reprogramming regulation techniques.

## Introduction

Neurological disorders represent a global health issue with inadequate therapeutic options (Rahman et al., 2022), ranking as the leading cause of disability and the second leading cause of death worldwide. According to Gooch et al. (2017), nearly 100 million Americans alone are affected by over 1000 neurological disorders, incurring an overall cost of $765 billion for prevalent conditions like Alzheimer’s disease (AD) and other dementias, Parkinson’s disease (PD), chronic low back pain, stroke, traumatic brain injury, migraine, and glioblastoma (Feigin et al., 2021).

Conventional treatments, however, offer limited efficacy, further compounding this crisis. Although existing pharmacological interventions primarily relieve symptoms, they fail to reverse the pathological process. For instance, 30% of PD patients develop complications within 2–3 years of long-term levodopa (L-3,4-dihydroxyphenylalanine; L-DOPA) treatment, and this rises to over 50% after 5 years, with L-DOPA-induced dyskinesia eventually developing in virtually all patients as the disease progresses (Poewe et al., 2017; Servillo et al., 2024). Similarly, tissue plasminogen activator for ischemic stroke is constrained by a narrow therapeutic window of 4.5 h, low utilization (<10%), and risks of hemorrhage or reperfusion injury, while also failing to promote neurorepair or prevent long-term disability (Moskowitz et al., 2010; Walie et al., 2026). Furthermore, the complex mechanisms underlying neurological diseases (Burns and Quinones-Hinojosa, 2021) mean that existing therapies, which often target a single molecule or pathway, struggle to provide comprehensive intervention. Although recent approvals of anti-amyloid-β (Aβ) antibodies such as lecanemab represent incremental progress, their clinical benefit remains modest and controversial, underscoring the need for fundamentally different therapeutic strategies (Sims et al., 2023; van Dyck et al., 2023). Thus, novel tools are essential for unraveling complex mechanistic networks and facilitating multi-target drug screening to address these limitations.

The discovery of induced pluripotent stem cells (iPSCs) by Takahashi and Yamanaka (Takahashi and Yamanaka, 2006) opened new avenues for patient-specific neuronal regeneration. However, iPSC-based approaches continue to face persistent challenges including tumorigenicity from residual pluripotent intermediates (Golpour and Prsian, 2025; Gong et al., 2019), complex multi-step differentiation protocols (Ebrahimi et al., 2019), and incomplete functional maturation of derived neurons (Tian et al., 2016). These limitations have driven the exploration of direct reprogramming as an alternative strategy that directly converts somatic cells from one lineage to another without passing through a pluripotent state (Davis et al., 1987; Wang H. et al., 2021). Since the seminal demonstration by Davis et al. (1987) that MyoD overexpression could convert fibroblasts into myoblasts, direct reprogramming has been achieved across multiple cell lineages using defined transcription factors, microRNAs, and small molecules (Abernathy et al., 2017; Ieda et al., 2010). Compared with conventional cell lines and primary neuronal cultures, which are experimentally accessible and scalable but limited in physiological relevance, direct reprogramming offers a more streamlined path to neuronal fate, with relatively rapid conversion kinetics and the unique potential for *in vivo* application (Puglisi et al., 2024; Wang G. et al., 2024). For neurological diseases, this approach holds particular promise because *in situ* conversion of endogenous glial cells into functional neurons could reduce tumorigenic risks, avoid exogenous cell transplantation, and allow newly generated neurons to mature within their native microenvironment.

Direct neuronal reprogramming is driven by proneural transcription factors such as ASCL1, NEUROG2, and NeuroD1, which initiate chromatin remodeling and activate neurogenic programs (Bocchi et al., 2022; Qin et al., 2017; Wapinski et al., 2013), while repressors like polypyrimidine tract-binding protein 1 (PTBP1) and RE1-silencing transcription factor (REST) normally block neuronal differentiation in non-neuronal cells (Ballas et al., 2005; Colasante et al., 2019; Xue et al., 2013). However, translating these mechanisms from *in vitro* to *in vivo* settings presents significant challenges. Notably, although PTBP1 knockdown has been reported to convert glial cells into dopaminergic neurons and ameliorate motor deficits in Parkinson’s disease models (Qian et al., 2020; Zhou et al., 2020), subsequent studies have raised concerns about potential artifacts from viral delivery systems and the need for rigorous lineage-tracing validation. These issues will be discussed in detail later in this review.

Encouragingly, direct neuronal reprogramming has already shown promising results across multiple neurological disease models. NeuroD1-mediated astrocyte-to-neuron conversion has been reported to improve motor and cognitive functions in ischemic stroke models (Chen et al., 2020); microRNA (miRNA)-based reprogramming has enabled patient-specific modeling of late-onset Alzheimer’s disease (Sun et al., 2024); and transcription factor cocktails have achieved functional retinal ganglion cell regeneration in retinal injury models (Todd et al., 2022; Xiao et al., 2021). These advances highlight the therapeutic potential of this approach, while also revealing critical challenges that must be addressed for clinical translation.

**FIGURE 1:**
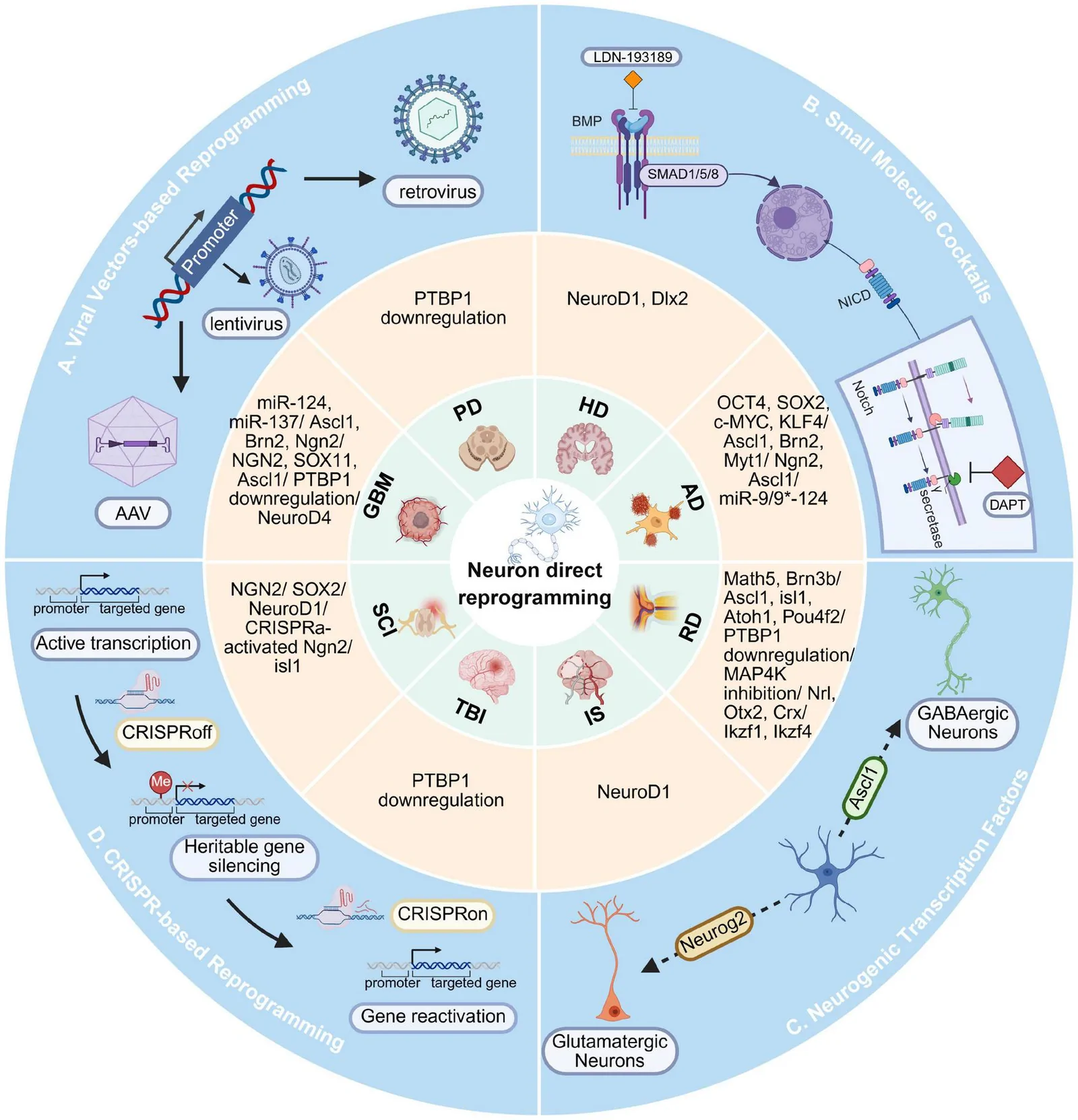
Overview of direct neuronal reprogramming applications for neurological disorders. The Figure provides an overview of the key induced transcription factors (TFs), microRNAs, and chemical molecules for direct neuronal reprogramming in the treatment of neurological disorders. It also outlines four major strategies for achieving neuronal conversion: **(A)** Viral vector-mediated neuronal direct reprogramming, **(B)** Chemical molecule-induced neuronal direct reprogramming, **(C)** Neurogenic TFs-induced neuronal direct reprogramming, **(D)** CRISPR-based reprogramming technologies. AD: Alzheimer’s disease; GBM: glioblastoma; HD: Huntington’s disease; IS: ischemic stroke; PD: Parkinson’s disease; RD: retinal degenerative diseases; SCI: spinal cord injury; TBI: traumatic brain injury; TFs: transcription factors. Created in BioRender. Chen, Y. (2026) https://BioRender.com/gva11xj.

In this review, we will discuss the current applications, dilemmas and prospects faced by direct neuronal reprogramming technology in neurological diseases (Figure 1).

## Methods of direct neuronal reprogramming

### Mechanistic insights

Direct neuronal reprogramming requires coordinated activation of neuronal regulatory networks, suppression of the donor-cell program, and acquisition of mature neuronal properties (Vierbuchen et al., 2010; Wapinski et al., 2013; Xue et al., 2013). At the initiation stage, proneural transcription factors such as ASCL1, NEUROG2, and NEUROD1 act as fate-instructive regulators, with ASCL1 functioning as a pioneer factor that engages neuronal regulatory elements in relatively inaccessible chromatin (Chanda et al., 2014; Heinrich et al., 2010). This early binding is followed by increased accessibility at neuronal enhancers and promoters and by recruitment of additional transcriptional and epigenetic regulators (Wapinski et al., 2017). Small molecules such as valproic acid, forskolin, and CHIR99021 can further facilitate reprogramming by modulating histone acetylation, cyclic adenosine monophosphate (cAMP) signaling, and Wnt/glycogen synthase kinase 3 beta (GSK3β) pathways, thereby improving the permissiveness of the starting cell state (Hu et al., 2015; Li et al., 2015; Pfisterer et al., 2016). At the same time, neuronal genes must be released from repression by circuits such as the PTBP1–miRNA–REST axis (Xue et al., 2016). However, conversion does not fully erase the donor cell’s molecular history; residual epigenetic and age-related signatures can affect maturation, disease phenotypes, and long-term stability (Mertens et al., 2015, 2021; Victor et al., 2018). After fate induction, brain-derived neurotrophic factor (BDNF), glial cell line-derived neurotrophic factor (GDNF), and neurotrophin-3 (NT-3) support survival, neurite growth, and electrophysiological maturation (Hu et al., 2015; Vierbuchen et al., 2010). *In vivo*, these cell-intrinsic processes are further modified by the local tissue environment, making lineage tracing and independent functional validation indispensable (Chen et al., 2022; Hoang et al., 2022; Wang L. L. et al., 2021).

### Inducing methods

Direct neuronal reprogramming depends on both the inducing cue and the method used to deliver it. Common inducing cues include transcription factors, small molecules, microRNAs, short hairpin RNAs, antisense oligonucleotides, and clustered regularly interspaced short palindromic repeats (CRISPR)-based regulators, while delivery platforms can be broadly classified into viral, physical, and chemical or biomaterial-based systems. Viral vectors are the most commonly used tools because they efficiently deliver transcription factors or RNA-based regulators; retroviral and lentiviral vectors are mainly used *in vitro* for stable transgene expression, whereas adeno-associated virus is widely used *in vivo* because of its relatively low pathogenicity and compatibility with cell-type-specific promoters (Chen et al., 2020; Vierbuchen et al., 2010). Non-viral physical methods, including electroporation, nanochannel electroporation, microfluidic mechanoporation, nano grooved substrates, and electrical stimulation, introduce reprogramming cargos through transient membrane permeabilization or biophysical stimulation, but are often limited by cell damage and variable efficiency (Gallego-Perez et al., 2016). Chemical and biomaterial-based systems, including lipid carriers, cell-penetrating peptides, synthetic cationic polymers, mesoporous silica nanoparticles, functionalized nanodots, hydrogels, and extracellular vesicles, provide safer and more controllable alternatives for delivering reprogramming factors (Chang et al., 2018; Connor et al., 2018). Although small molecules and CRISPR-based tools are often discussed as distinct inducing strategies, in practice they are often combined with these platforms to alter chromatin, signaling, or endogenous neurogenic genes (Qin et al., 2017; Zhou et al., 2020). The appropriate strategy therefore depends on the donor cell, desired neuronal subtype, experimental setting, duration of expression, genomic safety, and the controls available to verify cell origin (Wang L. L. et al., 2021).

### Experimental workflow

Experimental workflows generally proceed from donor-cell selection and isolation through induction, maturation, and validation; variation at any of these stages affects efficiency and reproducibility (Ambasudhan et al., 2011; Treutlein et al., 2016; Wapinski et al., 2013). The workflow begins with the selection of appropriate starting cells. Dermal fibroblasts are readily obtained and expanded, whereas peripheral blood cells, astrocytes, Müller glia, NG2-expressing glia, glioblastoma cells, and other disease-relevant populations require context-specific isolation and purification (Kraskovskaya et al., 2023; Saito et al., 2025; Sun et al., 2024). For *in vitro* or *ex vivo* studies using neural or glial starting populations, cell-type purity is particularly important, because contaminating endogenous neurons or mixed glial populations may lead to false-positive interpretation. Fluorescence-activated cell sorting, magnetic-activated cell sorting, immunopanning, reporter-based enrichment, and genetic labeling systems can therefore be used to improve donor-cell specificity; *in vivo* studies additionally require cell-selective promoters and independent lineage tracing (Chen et al., 2022; Hoang et al., 2022; Wang L. L. et al., 2021). After isolation, *in vitro* donor cells should be maintained under defined conditions, with passage number, seeding density, matrix coating, medium composition, and feeding schedule recorded. During induction, maintenance medium is commonly replaced with a chemically defined neurogenic medium containing N2 or B27 supplements, cAMP-elevating agents, ascorbic acid, forskolin, and neurotrophic factors (Giacomoni et al., 2025). Reprogramming cues and delivery platforms are then applied according to the selected experimental design, while factor combination, dosage, timing, promoter specificity, expression duration, and control settings should be carefully optimized (Kim J. et al., 2025; McCaughey-Chapman et al., 2024; Wapinski et al., 2017; Xue et al., 2013; Zhou et al., 2020). Conversion proceeds through progressive changes in morphology, neurite outgrowth, dendritic arborization, synaptic protein expression, and electrophysiological maturation rather than an instantaneous switch (Kajtez et al., 2025). Accordingly, neuronal identity should be examined at several time points using markers such as class III β-tubulin (TUBB3), microtubule-associated protein 2 (MAP2), neuronal nuclear antigen (NeuN), and synapsin, together with subtype and donor-cell markers that reveal incomplete conversion (Chen et al., 2022; Hoang et al., 2022; Wang L. L. et al., 2021).

A convincing result requires molecular, functional, and, for *in vivo* studies, lineage-based evidence. Immunostaining and transcriptomic profiling, particularly single-cell RNA sequencing, can confirm activation of neuronal programs, loss of donor-cell identity, and intermediate states along the conversion trajectory (Treutlein et al., 2016). Functional assays, including calcium imaging, multielectrode array recording, neurotransmitter release assays, and whole-cell patch-clamp electrophysiology, are needed to assess neuronal excitability and synaptic maturation (Ambasudhan et al., 2011; Kajtez et al., 2025; Kraskovskaya et al., 2023; Vierbuchen et al., 2010). For *in vivo* studies, however, functional neuronal properties alone do not prove cellular origin. Genetic lineage tracing, 5-bromo-2′-deoxyuridine (BrdU) or 5-ethynyl-2′-deoxyuridine (EdU) labeling, pre-labeling of endogenous neurons, trajectory analysis, and time-lapse imaging provide complementary ways to distinguish genuine conversion from viral leakage, reporter misexpression, injury-induced phenotypic change, or neuronal contamination (Chen et al., 2022; Hoang et al., 2022; Wang L. L. et al., 2021). In addition, three-dimensional cultures, spheroids, organoids, and microfluidic platforms can support longer maturation and more complex cell–cell interactions than conventional two-dimensional cultures (Giacomoni et al., 2025; Kajtez et al., 2025; Sun et al., 2024). Thus, standardized workflows from cell isolation to molecular, functional, and lineage-based validation are essential for improving reproducibility and advancing direct neuronal reprogramming toward disease modeling, drug screening, and regenerative therapy.

## Applications of direct neuronal reprogramming in neurological diseases

### Cell sources in direct reprogramming

The donor cell is not a neutral starting material: it affects conversion efficiency, residual molecular age, and the neuronal subtype that can be produced. Fibroblasts remain widely used because they are accessible and retain patient- and age-associated signatures. However, skin-derived neural crest precursors can account for a substantial fraction of neurons generated from some skin cultures, so donor-cell heterogeneity should be measured rather than assumed (Belair-Hickey et al., 2024). Endogenous glia serves different purposes *in vivo*: astrocytes are frequently targeted in the brain, whereas Müller glia is the principal retinal source (Portela-Lomba et al., 2024). Pericytes, oligodendrocyte precursor cells, and other glial populations have also demonstrated neuronal plasticity, although their reprogramming efficiency and lineage competence appear to be highly dependent on developmental origin and local microenvironment (Cates et al., 2025). Beyond conventional donor cells, increasingly accessible sources such as urine-derived cells and olfactory ensheathing cells are emerging as attractive alternatives because they can be obtained with minimal invasiveness while retaining efficient neuronal conversion capacity, broadening opportunities for patient-specific disease modeling and regenerative medicine (Nagai et al., 2024).

### Neurodegenerative diseases

Neurodegenerative diseases are the primary cause of cognitive and physical disability worldwide, affecting over 15% of the population (Feigin et al., 2020). They are debilitating conditions marked by the progressive deterioration of nerve cells in the brain and spinal cord, such as Alzheimer’s disease (AD), Parkinson’s disease (PD), retinal degenerative diseases (RDs), Huntington’s disease (HD), etc. These diseases can affect a person’s movement, cognition, and behavior, and often share underlying pathological mechanisms such as protein misfolding, synaptic dysfunction, neuroinflammation, and aging-related cellular stress. Because these pathological processes preferentially affect specific neuronal subtypes and circuits, direct neuronal reprogramming strategies should be matched to the vulnerable neuronal population, such as aging-featured patient-derived neurons for AD modeling (Herdy et al., 2022; Mertens et al., 2015; Sun et al., 2024), dopaminergic neurons for PD (Kim J. et al., 2025), retinal neurons for RDs (Todd et al., 2022), and striatal neurons for HD (McCaughey-Chapman et al., 2024). Critically, the mammalian central nervous system possesses limited regenerative capacity, making the neuronal loss largely permanent. Current treatments predominantly offer symptomatic relief and fail to halt or reverse the underlying neurodegenerative process, leading to significant disability and decreased quality of life. The unmet medical need drives the search for novel therapeutic strategies, including those aimed at replacing lost neurons to restore function, which makes direct neuron reprogramming a promising approach.

#### Alzheimer’s disease

Alzheimer’s disease is characterized by early cognitive deficits due to synaptic network dysfunction. Despite several conventional drugs targeting amyloid-beta (Aβ) or tau, these approaches have largely failed to alleviate such deficits, underscoring the critical need for novel therapeutic targets. Human neural stem cells (NSCs) offer a potentially unlimited source of different neural cell types for repairing the degenerated or injured brain. However, human adult NSCs persist in special niches of the brain and are inaccessible (Gage, 2000). Therefore, the *in vitro* generation and potential therapeutic applications of human induced neural progenitor cells (iNPCs) are of special interest. Zhang et al. (2019) reported that the Yamanaka factors, combined with a cocktail of four chemicals (SB431542, CHIR99021, VPA, and Forskolin, collectively known as SCVF), achieved reprogramming peripheral blood-derived mononuclear cells into iNPCs capable of over 25 serial passages and neurosphere formation, circumventing the challenges of obtaining brain tissues. Human iNPCs differentiate into mature neurons in AD mice brains, integrating the function of grafted human neurons into the synaptic networks of the host hippocampus. This leads to improved synaptic plasticity and enhanced cognitive abilities, indicating that restoring synaptic failures might offer new avenues for the development of novel treatments for AD (Zhang et al., 2019). However, the previous electrophysiological measurements have been limited on neurons transplanted in young rodents. The investigation into electrical activities of grafted human iNPCs within the brains of non-human primates, which are similar to humans, has been scarce. Feng et al. (2022), based on the Zhang’s study, further confirmed that human induced neural stem cells differentiated into neurons displaying long-term survival and electrophysiological activities similar to those of mature neurons in monkey brains. However, it remains unknown whether the established AD monkeys acquire cognitive deficits associated with AD, so it will still be impossible to test the therapeutic potential of human iNPCs in non-human primate models of AD (Feng et al., 2022; Yue et al., 2021).

Despite decades of research, AD remains a debilitating, progressive, and ultimately fatal dementia with no known cause aside from advanced age. While prevailing hypotheses of AD pathogenesis, such as Aβ deposition, tau tangles, and neuronal loss, have been extensively investigated, they fail to fully account for the age-related nonlinear trajectory of pathological escalation. The direct conversion of somatic cells into neurons provides a platform to study disease mechanisms in patient-specific neurons (Vierbuchen et al., 2010), thereby enabling precise dissection of cell-autonomous pathologies related to AD. Herdy et al. (2022) utilized the Lentiviral vector pLVXUbC-rtTA-Ngn2:2A: Ascl1 to deliver the core neurogenic transcription factors (TFs) Ngn2 and Ascl1, successfully converting AD-derived fibroblasts into functional induced neurons. They reported that induced neurons (iNs) display prominent senescence-associated phenotypes, including increased p16 expression and activation of the senescence-associated secretory phenotype. These senescent-like neurons drive neuroinflammation through IL-6/STAT3-mediated astrocyte activation, exacerbating the age-related deterioration in AD. The study shows that senotherapeutics, including senolytics such as navitoclax (ABT-263) that selectively eliminate senescent cells by inhibiting anti-apoptotic BCL-2 family proteins, could be a strategy for preventing or treating AD (Zhu et al., 2015). By generating iNs from AD patient fibroblasts, the study modeled post-mitotic neuronal senescence, a hallmark of AD brains, and finally identified neuronal senescence as a distinct pathogenic mechanism that may complement the classical Aβ- and tau-associated pathways (Herdy et al., 2022). Sun et al. (2024) deploy microRNA (miRNA)-based direct reprogramming, using MiR-9/9* and miR-124 to efficiently convert patient fibroblasts into Cholinergic neurons within a three-dimensional (3D) environment, successfully modeling Late-onset Alzheimer’s disease (LOAD) neuronal loss and tau tangles. The study identifies the molecular drivers underlying the senescence phenotypes in Herdy et al.’s (2022) AD neuron model, demonstrating that inhibiting age-associated retrotransposable elements in LOAD neurons mitigates Aβ deposition and neurodegeneration, thereby elucidating the previously unexplored Aβ-tau temporal interaction in this model. Meanwhile, they underscore the efficacy of modeling late-onset neuropathology of LOAD through high-efficiency miRNA-based neuronal reprogramming (Sun et al., 2024). By generating iNs from AD patient fibroblasts, both studies bypassed the epigenetic “rejuvenation” seen in iPSC-derived neurons, retaining donor-specific aging signatures. This enables precise modeling of age-associated neuronal senescence and retrotransposon activation in LOAD, offering a robust platform to identify novel therapeutic targets and develop personalized anti-aging interventions in AD research.

#### Parkinson’s disease

The primary cause of PD is the loss of dopamine-producing neurons in the brain. Dopamine is a neurotransmitter that plays a crucial role in regulating movement and emotional responses. The loss of these neurons leads to the motor symptoms associated with PD. Direct neuronal reprogramming offers a promising therapeutic strategy to address this neuronal loss by converting non-neuronal cells into functional dopaminergic neurons.

Polypyrimidine tract-binding protein 1 (PTBP1) is a key target in these efforts, which was initially identified as a regulator of alternative splicing and also plays various roles, including modulating mRNA metabolism, protein translation, and cell proliferation (Dai et al., 2022; Monzón-Casanova et al., 2020). In 2013, a research group reprogrammed primary mouse embryonic fibroblasts into functional neurons by downregulating PTBP1 expression, and demonstrated that this phenomenon is based on the relief of PTBP1-mediated blockage of miRNA action on multiple components of the REST complex, thereby releasing a large array of neuron-specific genes including ASCL1, MYT1, NEUROD1, and BRN2 (Xue et al., 2013, 2016) in non-neuronal cells. This converts a negative feedback loop into a positive feedback loop, causing reprogramming of non-neuronal cells toward the neuronal lineage cells. Furthermore, the suppression of PTBP1 results in a temporary increase in PTBP2 levels, a phenomenon observed during natural neurogenesis and essential for neuronal maturation (Xue et al., 2016). In 2020, this group and another published almost at the same time that knocking down PTBP1 can convert midbrain astrocytes into dopaminergic neurons in a chemically induced model of mouse PD to rebuild the nigrostriatal loop. Reinnervation of the striatum may promote restoration of dopamine levels and thereby improve motor deficits in PD (Guo et al., 2014; Qian et al., 2020). However, the possibility that alterations in the microenvironment leading to reduced neuroinflammation or neurotoxic glial cells loss contribute to therapeutic benefits following PTBP1 knockdown cannot be ruled out (McDowall et al., 2024).

Controversially, the replicability and specificity of *in vivo* PTBP1 downregulation were quickly questioned by other neuroscientists. One study demonstrated through rigorous lineage tracing that intracranial mouse astrocytes cannot be reprogrammed into neurons by down-regulating PTBP1, and that those putative astrocyte-converted neurons are actually endogenous neurons (Wang L. L. et al., 2021). In another study, Hoang et al. (2022) selectively deleted Ptbp1 in adult Müller glia and found that glial identity was maintained without neuronal conversion. A group further studied the reprogramming effect of down-regulation of PTBP1 on reactive astrocytes in a mouse Parkinson’s model, and did not find dopaminergic neurons originating from astrocytes (Chen et al., 2022).

Given the controversies and limitations of PTBP1-mediated *in vivo* reprogramming, especially for astrocytes, researchers have pursued alternative strategies. Recently, researchers demonstrated that electromagnetized Ti_3_C_3_T_×_ MXenes, serving as conductive biomaterial interfaces under low-frequency electromagnetic stimulation, enhance the direct reprogramming of human skin fibroblasts into induced dopaminergic neurons by promoting histone acetylation, chromatin accessibility, and dopaminergic neuronal maturation. This enhancement is achieved by activating histone acetylation under low-frequency electromagnetic fields, offering a promising advancement for PD therapy (Kim J. et al., 2025).

#### Retinal degenerative diseases

Retinal degenerative diseases, including age-related macular degeneration, retinitis pigmentosa, diabetic retinopathies, glaucoma, and numerous hereditary retinal disorders (Wan and Goldman, 2016), currently lack a cure. Restoring vision loss caused by the irreversible degeneration of retinal neurons is a major challenge in ophthalmology. Cell reprogramming for retinogenesis presents itself as a promising technique for addressing this challenge.

A primary focus is the direct conversion of endogenous Müller glia (MG) into new retinal ganglion cells (RGCs) as a potential strategy for treating optic neuropathies such as glaucoma (Soucy et al., 2023). Previous studies have demonstrated that expressing specific combinations of TFs, including Math5 and Brn3b (Xiao et al., 2021), as well as Ascl1, Pou4f2, Islet1, and Atoh1 (Todd et al., 2022), can successfully reprogram mature mouse MG into RGCs. Beyond TF cocktails, other approaches like knockdown of *Ptbp1* or inhibition of MAP4K through treatment with DMX-5804 have also prompted the reprogramming of MG into RGCs (Zhang et al., 2023; Zhou et al., 2020). A consistent finding across these studies is that the reprogrammed RGCs are capable of extending long axons and innervating appropriate brain targets. Notably, an alternative pathway involving ectopic expression of Oct4, Sox2, and Klf4 has also been employed to reprogram mouse RGCs, uniquely highlighting the role of DNA demethylases TET1 and TET2, suggesting that accessing youthful epigenetic information can promote tissue regeneration (Lu et al., 2020).

Direct reprogramming of MG is also being explored to regenerate other crucial retinal interneurons. Bipolar cells receive electrical signals from photoreceptors and convey the extracted visual information to RGCs, while amacrine cells contribute to complex visual processing (Liou et al., 2020). Both are susceptible to damage from conditions like Diabetic Retinopathy, which can lead to visual defects. Researchers have found that MG-specific overexpression of Ascl1, together with a histone deacetylase inhibitor, facilitates MG transformation into amacrine and bipolar cells (Jorstad et al., 2017). Disruption of nuclear factor I TFs induces MG to proliferate and generate amacrine-like and bipolar-like cells in adult mice after injury (Hoang et al., 2020). These neuronal reprogramming strategies targeting MG hold therapeutic potential for restoring visual function in Diabetic Retinopathy.

Furthermore, given that photoreceptor degeneration directly drives irreversible vision loss in diseases like retinitis pigmentosa (Liou et al., 2020), regenerating these light-sensing cells from MG is a key focus. Yao et al. (2018) showed that following gene transfer of β-catenin, cell-cycle-reactivated MG can be reprogrammed to generate rod photoreceptor with TFs Otx2, Crx, and Nrl. In 2023, another group found that cone-like cells were achieved by combined expression of Ikzf1 and Ikzf4 (Boudreau-Pinsonneault et al., 2023), offering potential avenues to restore photoreceptive capacity in degenerative retinopathies.

In recent years, the application of direct reprogramming technology has expanded from endogenous glial cells to other cell populations within the eye that possess powerful regenerative potential. Among these, the stem and progenitor cells within the ciliary marginal zone (CMZ) were identified and leveraged for novel *in situ* direct reprogramming strategies owing to their intrinsic neurogenic characteristics. Marcucci et al. (2016) provided crucial evidence that the mammalian embryonic CMZ could generate retinal ganglion cells and identified Cyclin D2 as a critical regulator for their proliferation and differentiation. This discovery provided a molecular basis for the *in situ* reprogramming of CMZ cells and demonstrated the regenerative potential of the mammalian CMZ to restore the entire retina. Liu et al. (2025) addressed the long-standing debate about the regeneration of the human retinal CMZ by identifying distinct human neural retinal stem-like cells within the CMZ. These cells have great potential for *in situ* retinal regeneration and visual functional recovery in degenerative models, which could serve as a novel cell source for regenerative therapies, bypassing the need for exogenous transplantation and targeting retinal degenerative diseases.

#### Huntington’s disease

Huntington’s disease is a progressive, inherited neurodegenerative genetic disorder that affects muscle coordination and leads to cognitive decline and psychiatric symptoms. It is caused by a mutation in the huntingtin gene, which leads to the production of an abnormal form of the huntingtin protein. Crucially, there is currently no cure for HD.

In addressing this challenge, direct cell reprogramming technology has emerged as a promising avenue for both studying disease mechanisms and developing potential therapies. Liu et al. (2014) pioneered the direct reprogramming of HD patient fibroblasts into striatal neuron-like cells via PTB knockdown. These converted cells successfully recapitulated key HD neuropathological features, including mutant huntingtin aggregation, neuritic abnormalities, and increased cell death, thereby establishing a disease-specific model for mechanistic and therapeutic exploration (Liu et al., 2014). Victor et al. (2018) directly converted HD patient fibroblasts into striatal medium spiny neurons using miR-9/9*-124 combined with striatal transcription factors (CTIP2, DLX1, DLX2, MYT1L), building upon their earlier methodology. Crucially, these directly reprogrammed MSNs retained age-associated disease phenotypes, including mutant huntingtin aggregation and spontaneous neurodegeneration, which were absent in iPSC-derived neurons from the same patients. This demonstrates a key advantage of direct reprogramming for modeling age-dependent neurodegenerative diseases (Victor et al., 2018). Subsequently, Monk et al. (2021) utilized a non-viral approach based on chemically modified mRNA to directly reprogram adult HD patient fibroblasts into induced neural precursor cells that differentiated into DARPP32+ striatal neurons. These HD neurons exhibited ubiquitinated polyglutamine aggregates, impaired neuronal maturation with altered neurite morphology and depolarized resting membrane potentials, and reduced BDNF levels that correlated with CAG repeat length and disease onset age, establishing a clinically relevant model for investigating HD pathogenesis and drug screening (Monk et al., 2021). In 2022, a study modeled age-dependent neurodegeneration in HD using directly reprogrammed striatal medium spiny neurons (MSNs) from patient fibroblasts, revealing that aging-associated upregulation of miR-29b-3p promotes neuronal death by impairing autophagy, and that modulating this pathway can rescue HD-MSN degeneration (Oh et al., 2022). A group reports that AAV-mediated ectopic expression of NeuroD1 and Dlx2 TFs can reprogram striatal astrocytes into GABAergic neurons with high efficiency (>80%) in a mouse model of HD, thereby prolonging lifespan and improving motor function. But this study is also subject to the same skepticism as downregulating PTBP1 to initiate astrocyte-to-neuron conversion (Wu et al., 2020). There are currently no reports on the application of neuronal reprogramming technology in other neurodegenerative diseases. In addition, based on the above-mentioned controversy of astrocyte-to-neuron conversion *in vivo*, we will discuss how to rigorously define successful reprogramming later in this review. McCaughey-Chapman et al. (2024) demonstrated that direct reprogramming generates human induced lateral ganglionic eminence precursors (hiLGEPs) from adult human dermal fibroblasts using SOX2 and PAX6 mRNA. These hiLGEPs differentiate into functional striatal neurons that restore motor function in a HD rat model, presenting a promising cell replacement strategy. By utilizing a non-viral delivery system for the generation of hiLGEPs, this research successfully bypassed potential contamination issues linked to viral vectors.

### Nerve damaging diseases

Neurological disorders and conditions such as stroke, traumatic brain injury, and spinal cord injury (SCI) often result in nerve damage and impairments in the nervous system functioning, leading to severe symptoms such as paralysis, weakness, sensory disturbances, and cognitive deficits. A major challenge in the chronic phase of these diseases is that conventional postoperative treatments for these diseases are difficult to substantially change the patient’s prognosis.

During the chronic phase of these diseases, glial cells, particularly astrocytes, proliferate significantly and eventually form glial scars at the lesion site. The dense structure of glial scars hinders the growth and extension of regenerating nerves, and releases inhibitory factors or inflammatory factors that disrupt nerve regeneration. This lesion-associated accumulation of reactive glia provides a pathological basis for *in situ* reprogramming, because reactive astrocytes near ischemic or traumatic lesions can be targeted for neuronal conversion (Chen et al., 2020; Liu J. et al., 2024), while NG2 glia represent another local cellular source for neuronal regeneration after SCI (Tai et al., 2021). Unlike iPSC-based transplantation, direct reprogramming bypasses *ex vivo* differentiation and grafting steps, allowing newly generated neurons to arise close to the lesion and potentially integrate into existing host circuits, as suggested in ischemic stroke and SCI models. Neuronal reprogramming technology could be used to reprogram the proliferating astrocytes into functional neurons to rebuild the neural network. A team reported that using NeuroD1 AAV vector can efficiently mediate the *in situ* conversion of astrocytes into neurons. In the study, they used patch-clamp techniques to demonstrate that the converted neurons are functional, and revealed the axonal growth of the converted neurons and their integration into the normal neural network process using anterograde and retrograde tracing. Mice model of ischemic stroke showed significant improvements in both motor and cognitive functions after receiving treatment with this technology (Chen et al., 2020). Furthermore, in a recent study on traumatic brain injury (TBI), astrocyte-targeting peptide P1 and GFAP promoter were combined to specifically knock down PTBP1 in astrocytes, thereby initiating direct neuronal reprogramming. This study provides a novel gene delivery vector for studying neuronal reprogramming and its potential applications in TBI (Liu J. et al., 2024).

Beyond stroke and TBI, direct neuronal reprogramming is also being explored in spinal cord injury models. TFs such as NGN2, SOX2, NeuroD1, and their combination with neurotrophic factors (BDNF-NOG) have been applied to convert spinal cord astrocytes or NG2 glial cells into neurons. Zhou et al. (2021) used the CRISPRa tool to activate the endogenous genes *Ngn2* and *Isl1*, reprogramming mouse spinal cord astrocytes and embryonic fibroblasts into motor neurons with typical morphology and electrophysiological activity. Tai et al. (2021) demonstrated that Sox2 could convert NG2 glial cells into both excitatory and inhibitory spinal neurons, and these iNs were capable of establishing synaptic connections with both ascending and descending spinal pathways. Furthermore, this technique reduced glial scarring and promoted functional recovery after SCI (Tai et al., 2021).

While these preclinical findings across different central nervous system (CNS) injury models are highly promising, it is important to note that aspects of *in vivo* glial-to-neuron conversion, particularly regarding conversion efficiency, long-term functional integration, and the exact lineage origin in some contexts, remain areas of active research and discussion in the field. Nevertheless, the potential of reprogramming glial cells into functional neurons represents a significant step forward in addressing the limited regenerative capacity of the injured CNS.

### Central nervous system tumors

Tumors of the CNS often lead to severe clinical symptoms, such as headaches, intracranial hypertension, paralysis, and seizures. Glioblastoma (GBM) is one of the most malignant among them, and despite comprehensive treatment involving surgery and radiochemotherapy, the median survival period for patients does not exceed 2 years. Therefore, the development of new treatment methods for GBM is urgently needed. Given that GBM originates from neural glial cells, which possess a degree of plasticity and have been shown capable of directly converting into neurons *in situ*. So, could this cellular plasticity be used to reprogram highly malignant GBM cells into terminally differentiated neuron-like cells, thereby blocking their rapid proliferation and even restoring damaged neural function? In this context, direct reprogramming is not intended as classical neuronal replacement, but rather as a cell-fate-based anticancer strategy that redirects malignant GBM cells toward less proliferative and more differentiated states (Jiang et al., 2024), or reshapes tumor-supportive immune cells toward more antitumor phenotypes (Fermi et al., 2023; Liu T. et al., 2024). Compared with iPSC-based approaches, this strategy avoids pluripotent intermediates and directly targets the malignant cellular state within the tumor microenvironment, which is more consistent with the pathophysiology of GBM.

Early studies demonstrated that modulating specific factors could induce neuronal characteristics in GBM cells. In 2008, a group discovered through quantitative RT-PCR that the overexpressing miR-124 and miR-137, which are significantly decreased in GBM cells, successfully induced GBM cells to acquire a neuronal-like cell phenotype, and significantly slowed the growth rate of these converted tumor cells (Silber et al., 2008). Building on such initial findings, researchers explored the use of TFs to drive this conversion more efficiently. Zhao et al. (2012) further discovered that the TFs Ascl1, Brn2, and Ngn2 could reprogram GBM cell lines U251 and U87 into neuron-like cells with functional action potentials, thereby inhibiting their rapid proliferation. While this achieved a notable conversion efficiency of 20%–40%, which was much higher than that of primary human fibroblasts under the same conditions (suggesting glioma cells may have a more active transcriptional network and are more easily induced into neurons), achieving complete elimination of tumor cells requires even higher efficiency. Su et al. (2014) reported that the combined expression of NGN2 and SOX11 converted more than 95% of virus-transduced human glioma cells into neuron-like cells, which predominantly exhibited an excitatory neuronal phenotype and generated action potentials. Subsequently, Cheng et al. (2019) showed that ASCL1 alone converted approximately 94% of human glioma cells into excitatory and inhibitory neuron-like cells. Our team has also demonstrated that knocking down PTBP1 can convert PTEN-mutated GBM cells into neuron-like cells through the UNC5B receptor, and NeuroD4 overexpression can convert GBM cells into neuron-like cells via the SLC7A11-GSH-GPX4 axis. More broadly, NeuroD1-mediated direct reprogramming using AAV has been shown to convert GBM cells into post-mitotic neurons, suppressing tumor progression (Jiang et al., 2024).

In addition, strategies integrating direct cellular reprogramming with tumor immunotherapy are being developed to improve GBM cell elimination. Fermi et al. (2023) found that GW2580-mediated direct reprogramming of glioblastoma-associated macrophages from immunosuppressive M2 to proinflammatory M1 phenotype enhances antitumor T-cell responses and directly suppresses tumor growth, offering a novel therapeutic strategy for GBM. A different but complementary strategy focuses on reprogramming the tumor cells themselves to become immune-stimulatory. Liu T. et al. (2024) utilized machine learning-guided direct reprogramming to convert GBM cells into induced dendritic cell-like antigen-presenting cells, developing a conversion immunotherapy that synergized with PD1 decoy therapy to remodel the immunosuppressive microenvironment, activate tumor-specific CD8+ CTLs, and achieve robust tumor killing and prolonged survival in preclinical models.

The essence of these direct reprogramming strategies for GBM lies in inducing a fundamental cell fate change to combat the tumor. Whether converting malignant GBM cells into non-proliferating neuron-like cells or reengineering the tumor microenvironment by reprogramming immune cells, it is greatly different from the conventional research topic of inhibiting or killing tumor cells. This technology offers a gentler and potentially more precise way to target residual tumor cells after surgery, holding promise for novel clinical treatment approaches for GBM.

### Other neurological diseases

Direct neuronal reprogramming has also been used to model diseases outside the major categories above. Fibroblasts from patients with amyotrophic lateral sclerosis (ALS) or frontotemporal dementia (FTD) have been converted into motor or cortical neurons that retain disease-associated phenotypes (Bauer et al., 2019; Liu et al., 2016). Motor neurons generated from patients with spinal muscular atrophy showed impaired survival and neurite development (Zhang et al., 2017). Moreover, autism-associated NLGN3 mutations produced specific synaptic defects in directly converted neurons (Chanda et al., 2013), while Rett syndrome patient-derived neuron-like cells revealed MECP2-dependent transcriptomic and metabolic alterations (Karaosmanoglu et al., 2024). These studies extend direct conversion beyond cell replacement by providing patient-specific models that preserve genetic and, in many cases, age-related disease features.

We systematically summarized the methods of direct neuronal reprogramming for various diseases and their experimental outcomes in Table 1.

**TABLE 1:** Representative direct neuronal reprogramming strategies for neurological diseases.

Disease category	Disease	Starting cell type	Reprogramming factor in action	Reprogramming cofactor	Target cell type	Experimental outcome	Functional outcome	Ref.
Neurodegenerative diseases	PD^1^	Midbrain astrocytes	Downregulate ptbp1^2^	microRNAs targeting REST^3^ complex components	DAn^4^	0% astrocyte-originated DAn4	No functional recovery observed; no astrocyte-derived dopaminergic neurons detected in 6-OHDA PD mouse model	Chen et al., 2022
Neurodegenerative diseases	PD	Striatal astrocytes (*in vivo*)	CRISPRa-activated Ascl1, Lmx1a, Nr4a2	AAV-based intein-split-dCas9 activator system	GABAergic neurons	~12% GFP+/NeuN+ induced neurons	Induced GABAergic neurons functionally integrated into striatal circuits; significant amelioration of voluntary motor behavior in 6-OHDA PD mouse model; single-cell transcriptome confirmed neuronal identity	Giehrl-Schwab et al., 2022
Neurodegenerative diseases	PD	Human skin fibroblasts (*in vitro*)	Ascl1, Nurr1, Lmx1a	Electromagnetized Ti_3_C_2_T_×_ MXenes under low-frequency EMF	iDA^5^ neurons	Enhanced TH+/TUJ1+ neuron yield	Activated histone acetylation; enhanced dopaminergic neuron generation with superior electrical conductivity and biocompatibility; promising for PD therapy	Kim J. et al., 2025
Neurodegenerative diseases	AD^6^	PB-MNCs^7^	OCT4, SOX2, c-MYC, KLF4	SCVF^8^	iNPCs^9^	82% TUJ1+, 65% MAP2	Improved hippocampal synaptic plasticity and rescued spatial cognitive deficits in AD mice	Zhang et al., 2019
Neurodegenerative diseases	AD	Astrocytes from Dbh-2a-mCherry mice	Ascl1, Phox2b, AP-2α, Gata3, Hand2, Nurr1, Phox2a	/	iNA neurons^10^	8.8% TH+ mCherry+ neurons	Converted neurons exhibited spontaneous and evoked action potentials; norepinephrine release detected *in vitro*	Li et al., 2019
Neurodegenerative diseases	AD	AD-derived fibroblasts	Ngn2 and Ascl1,	/	iNs^11^	100% iNs	Identified senescence-associated secretory phenotype and IL-6/STAT3-mediated astrocyte activation in AD iNs	Herdy et al., 2022
Neurodegenerative diseases	AD	Mouse embryonic and postnatal fibroblasts	Ascl1, Brn2, and Myt1l	/	iNs	1.8%–7.7% MEF^12^ and TTF^13^ iNs	iNs expressed functional glutamate and GABA receptors; generated action potentials and formed functional synapses *in vitro*	Vierbuchen et al., 2010
Neurodegenerative diseases	AD	ADAD^14^/LOAD^15^-affected human fibroblasts	miR-9/9*-124	NEUROD2 and MYT1L	LOAD neurons	90% NCAM1+/MAP2+/TAU+ /TUBBIII+, >80% NEUN+ cells	Successfully modeled LOAD tau tangles and neuronal loss in 3D culture; identified retrotransposable element activation as molecular driver; demonstrated that inhibiting retrotransposons mitigated Aβ deposition	Sun et al., 2024
Neurodegenerative diseases	RDs^16^	MG^17^	Math5 and Brn3b	/	RGCs^18^	25.0% Brms+ RGCs, 16.2% Brn3a+ RGCs, 6.1% Tfap2a/2b+ amacrine cells	Regenerated RGCs extended axons into the optic nerve and projected to correct brain targets; partial restoration of pupil light reflex	Xiao et al., 2021
Neurodegenerative diseases	RD	MG	IPA^19^	The Atoh class of TFs^20^	Ganglion-like cells	80% HuC/D	Regenerated ganglion-like cells expressed mature RGC markers and extended axons; however, long-term functional integration was not fully assessed	Todd et al., 2022; Todd et al., 2021
Neurodegenerative diseases	RD	MG	Inhibit MAP4K4/6/7	DMX-5804	RGCs	54% EdU+/NeuN+/tdTomato+ cells	Regenerated RGCs extended axons into the optic nerve; improved pupil light reflex and optomotor response in NMDA injury model	Zhang et al., 2023
Neurodegenerative diseases	RD	MG	AAV-GFAP-CasRx- Ptbp1	/	RGCs	>50% Brn3a+/Rbpms+/ tdTomato+ cells	Demonstrated symptom alleviation across multiple neurological disease models (PD and retinal degeneration); converted neurons showed electrophysiological properties consistent with mature neurons	Zhou et al., 2020
Neurodegenerative diseases	RD	MG	Ikzf1/Ikzf4	/	iONL^21^	78% Rxrg+ cells	Ikzf1/Ikzf4 combination generated cone-like cells in the outer nuclear layer; photoreceptor identity confirmed by marker expression	Boudreau-Pinsonneault et al., 2023
Neurodegenerative diseases	RD	MG	Ascl1	Trichostatin-A^22^	Amacrine and bipolar cells	3.5% Pax6+/HuC/D+ cells, 17% Cabp5+ cells	Reprogrammed amacrine and bipolar cells showed light-responsive electrophysiological activity; functional integration demonstrated by multielectrode array recording	Jorstad et al., 2017
Neurodegenerative diseases	RD	MG	Nfia/b/xlox/lox	/	Bipolar cells, Amacrine cells	8.5% CRX+ neurons, 6.1% HuC/D+ cells	Disruption of NFI transcription factors induced MG proliferation and neurogenesis after injury; identified conserved gene regulatory networks controlling vertebrate retinal regeneration	Hoang et al., 2020
Neurodegenerative diseases	RD	MG	Otx2, Crx, and Nrl	ShH10-GFAP-β -catenin, a 2.1 kb rhodopsin promoter	Rod photoreceptor	97.4% Rhodopsin-tdTomato+ cells	De novo generated rod photoreceptors were light-responsive and integrated into existing retinal circuitry; partial restoration of visual function demonstrated by pupil light reflex and visual behavior tests	Yao et al., 2018
Neurodegenerative diseases	HD^23^	Striatal astrocytes	NeuroD1 and Dlx2 (ectopic expression via AAV^24^)	AAV delivery system	GABAergic^25^ neurons	>50% DARPP^26^32+ neurons	Extended lifespan and improved motor coordination in R6/2 HD mouse model	Wu et al., 2020
Neurodegenerative diseases	HD	Adult fibroblasts	miR-9/9*-124	CTIP2, DLX1, DLX2 and MYT1L	Striatal MSNs	~70%–80% MAP2+/TUBB3+ neurons; of which 90% GABA+, 70% DARPP32+	Converted MSNs persisted >6 months after transplantation in mouse brain; exhibited membrane properties equivalent to native MSNs; extended projections to anatomical targets of MSNs	Victor et al., 2014
Neurodegenerative diseases	HD	HD patient fibroblasts (CAG 40, 43, 44)	miR-9/9*-124	CTIP2, DLX1, DLX2 and MYT1L	Striatal MSNs	TUBB3+, GABA+, DARPP32+ neurons generated at comparable efficiency to controls (no significant difference, *p* > 0.97)	HD-MSNs retained age-associated disease phenotypes including mHTT aggregation, spontaneous neurodegeneration, electrophysiological dysfunction; revealed age-dependent transcriptomic signatures absent in iPSC-derived neurons; HD mutation did not impair conversion itself	Victor et al., 2018
Neurodegenerative diseases	HD	Adult HD patient fibroblasts	SOX2, PAX6 (chemically modified mRNA)	/	DARPP32+ striatal neurons	52–54% of TUJ1+ neurons co-expressed DARPP32 in HD lines	Subpopulation of HD neurons contained ubiquitinated polyQ aggregates; altered neurite morphology, more depolarized resting membrane potentials; reduced BDNF protein correlated with CAG repeat length and earlier disease onset age	Monk et al., 2021
Nerve damaging diseases	IS^27^	Astrocytes	NeuroD1 (AAV)	hGFAP::Cre/FLEX-CAG::NeuroD1	Neurons	74.3% recovery	Significant improvement in both forelimb reaching task and fear conditioning in IS mice; converted neurons showed action potentials and integrated into local neural circuits	Chen et al., 2020
Nerve damaging diseases	TBI^28^	Astrocytes	AAV9P1- shPTBP1	A1 peptide^29^/GFAP promoter	Neurons	55.4% NeuN+ cells	AAV9P1 dual-targeting system specifically knocked down PTBP1 in astrocytes; neuronal conversion confirmed in TBI mouse model but behavioral recovery under investigation	Liu J. et al., 2024
Nerve damaging diseases	SCI^30^	NG2+ glia cells	SOX2	/	DcX+ cells	64% DcX+/GFP+ cells	Reduced glial scarring; induced neurons formed synaptic connections with ascending and descending spinal pathways	Tai et al., 2021
Nerve damaging diseases	SCI	Spinal cord astrocytes	CRISPRa-activated Ngn2/Isl1	dCas9-VP64/ sgRNA	iMN^31^	70% ChAT+/Tuj1+ cells	CRISPRa-induced motor neurons exhibited typical morphology and electrophysiological activity	Zhou et al., 2021
Nerve damaging diseases	SCI	MEF				64.3% ChAT+/Tuj1+ cells		
Nerve damaging diseases	SCI	Spinal cord astrocytes (*in vivo*)	NeuroD1 and Ngn2	BrdU tracking of cell regeneration	Neurons	Significant increase in DCX+/NeuN+ cells at injury site (*p* < 0.0001)	Improved spinal nerve conduction (*p* < 0.0001); improved motor function and sensory function; enhanced blood-spinal cord barrier integrity; reduced TGF-β-mediated glial scarring	Talifu et al., 2024
Central nervous system tumors	GBM^32^	human GBM stem cells	miR-124/miR-137 (overexpression)	/	Neuron-like cells	59% Tuj1+ cells	Significantly slowed proliferation rate of GBM stem cells; induced G1 cell cycle arrest and neuronal differentiation	Silber et al., 2008
Central nervous system tumors	GBM	U251/U87 GBM cell lines	Ascl1+Brn2+Ngn2 (ectopic expression)	/	Neuron-like cells	A conversion efficiency of 20%–40%	Converted neuron-like cells generated functional action potentials; inhibited rapid proliferation of U251/U87 GBM cell lines	Zhao et al., 2012
Central nervous system tumors	GBM	GBM cells	NGN2+SOX11+ ASCL1 (ectopic expression)	/	Neuron-like cells	A conversion efficiency of >90%	Neuron-like cells generated action potentials and differentiated into excitatory, inhibitory, and motor neuron subtypes; significant inhibition of glioma growth *in vivo*	Cheng et al., 2019
Central nervous system tumors	GBM	PTEN-mutated GBM cells	PTBP1 knockdown	UNC5B receptor	Neuron-like cells	94.75%–95.51% TUJ1+ cells, 91.41%–91.55% MAP2+ cells	PTBP1 knockdown induced neural differentiation of PTEN-mutated GBM cells through UNC5B receptor; suppressed tumor cell proliferation	Wang K. et al., 2022
Central nervous system tumors	GBM	GBM cells	NeuroD4 (ectopic overexpression)	SLC7A11-GSH-GPX4 signaling axis	Neuron-like cells	84.8%–92.8% TUJ1+ cells, 75.8%–94.1% MAP2+ neuron-like cells	NeuroD4-mediated conversion suppressed GBM cell proliferation via SLC7A11-GSH-GPX4 antioxidant axis; induced ferroptosis-like cell death in remaining tumor cells	Wang et al., 2023
Central nervous system tumors	GBM	GBM cells	AAV-ND1	/	Post-mitotic neurons	A conversion efficiency of 50%	AAV-ND1 converted GBM cells into post-mitotic neurons, halting tumor progression; demonstrated tumor suppression *in vivo*	Jiang et al., 2024
Central nervous system tumors	GBM	GAMs^33^ (M2)	GW2580	/	GAMs (M1)	Suppressed tumor growth	GW2580 reprogrammed M2 into M1 glioblastoma-associated macrophages, enhancing antitumor T-cell responses and directly suppressing GBM growth in patient-derived models	Fermi et al., 2023
Central nervous system tumors	GBM	GBM cells	PU.1/IRF8/ID2/BATF3	dP-mF3	iDC-APC^34^s	A conversion efficiency of 60%–70%	Machine learning-guided conversion of GBM cells into dendritic cell-like APCs; synergized with PD1 decoy therapy to activate tumor-specific CD8+ CTLs; prolonged survival in preclinical models	Liu T. et al., 2024
Central nervous system tumors	GBM		PU.1+IKZF1	/		A conversion efficiency of 30%–70%		

^1^PD: Parkinson’s disease;

^2^ptbp1: polypyrimidine tract-binding protein 1;

^3^REST: RE1-silencing transcription factor;

^4^DAn: dopaminergic neurons;

^5^iDA: Induced dopaminergic;

^6^AD: Alzheimer’s disease;

^7^PB-MNCs: peripheral blood mononuclear cells;

^8^SCVF: SB431542, CHIR99021, VPA, and Forskolin;

^9^iNPCs: induced neural progenitor cells;

^10^iNA neurons: induced noradrenergic neurons;

^11^iNs: induced neurons;

^12^MEF: mouse embryonic fibroblast;

^13^TTF: tail tip fibroblast;

^14^ADAD: Alzheimer’s Disease and Associated Disorders;

^15^LOAD: late-onset Alzheimer’s disease;

^16^RDs: retinal degenerative diseases;

^17^MG: Müller glia;

^18^RGCs: retinal ganglion cells;

^19^IPA: Islet1, Pou4f2, Ascl1;

^20^TFs: transcription factors;

^21^iONL: Induced Outer Nuclear Layer;

^22^TSA: Trichostatin-A;

^23^HD: Huntington’s disease;

^24^AAV: adeno-associated virus;

^25^GABAergic: gamma-aminobutyric acidergic;

^26^DARPP: dopamine and camp regulated phosphoprotein;

^27^IS: ischemic stroke;

^28^TBI: traumatic brain injury;

^29^A1 peptide: astrocyte-targeting peptide P1;

^30^SCI: spinal cord injury;

^31^iMN: induced motor neuron;

^32^GBM: glioblastoma;

^33^GAMs: Glioma-Associated Microglia;

^34^iDC-APC: Interdigitating Dendritic Cells-Antigen Presenting Cell.

### Controversies and issues

Direct neuronal reprogramming has generated considerable excitement for its potential to treat neurological diseases by converting endogenous glial cells into functional neurons *in situ*. However, the field has been confronted with critical questions regarding the reliability of certain *in vivo* conversion claims. These concerns stem from both biological ambiguities, such as cellular heterogeneity, residual epigenetic memory, and incomplete neuronal maturation, and technical limitations, including poor reproducibility and insufficient methodological standardization. Beyond these general bottlenecks, one of the most prominent controversies specifically concerns the reliability of AAV-mediated delivery of NeuroD1 or PTBP1 knockdown. Rather than undermining the entire field, it has served as invaluable lessons, driving the establishment of more rigorous experimental standards and a deeper understanding of the technical pitfalls inherent to *in vivo* reprogramming.

### The PTBP1 controversy

The initial reports that PTBP1 knockdown could convert midbrain astrocytes into dopaminergic neurons and reverse motor deficits in Parkinson’s disease models generated tremendous interest (Guo et al., 2014; Qian et al., 2020). The underlying mechanism was compelling: relieving PTBP1-mediated repression of miRNA circuits targeting the REST complex would derepress neuronal gene programs, converting a negative feedback loop into a positive one and thereby redirecting non-neuronal cells toward a neuronal fate (Xue et al., 2013, 2016). However, these findings were rapidly challenged by independent groups employing more stringent lineage-tracing approaches. Wang L. L. et al. (2021) demonstrated that, following injection of NeuroD1-mCherry AAV, reporter-positive neurons appeared in the experimental region but showed no evidence of having passed through an immature developmental stage, nor did neuronal density increase in the injection area. These observations were inconsistent with genuine *de novo* neurogenesis originating from glial precursors (Wang L. L. et al., 2021). When they pre-labeled proliferating reactive astrocytes with BrdU prior to AAV-mediated conversion, the resulting NeuN-positive neurons were largely devoid of BrdU incorporation, directly contradicting the conclusion that these neurons originated from dividing reactive astrocytes. Furthermore, using the Cre-loxP system to permanently mark astrocytes, they found that NeuroD1 failed to convert genetically traced astrocytes into neurons (Wang L. L. et al., 2021). Consistent with these findings, Hoang et al. (2022) reported no astrocyte-derived neurons in astrocyte-specific PTBP1 deletion mice, and Chen et al. (2022) detected no dopaminergic neurons of astrocytic origin in a 6-OHDA Parkinson’s disease model following PTBP1 repression.

### AAV leakage

The convergent evidence from these studies points to AAV delivery system leakage as the primary source of false-positive results. To definitively demonstrate this, Wang L. L. et al. (2021) pre-labeled cortical neurons with rAAV2-retro-CAG-GFP before performing NeuroD1-mediated astrocyte reprogramming in the same area. A substantial proportion of the resulting NeuN-positive “converted” neurons were GFP-positive, confirming that they were pre-existing endogenous neurons that had been ectopically transduced by the AAV vector rather than newly generated from glial cells (Wang L. L. et al., 2021). This finding has profound implications. Studies reporting successful *in vivo* glia-to-neuron conversion that relied solely on reporter gene expression as evidence of conversion, without independent lineage-tracing verification, must be interpreted with extreme caution.

This artifact also raises a perplexing question: if genuine neuronal conversion is not occurring, why do so many animal models show improvements in neurological function following these interventions? While the micro-lesion effect of stereotactic injection, analogous to the transient symptomatic relief observed during deep brain stimulation electrode placement, might contribute, this is typically controlled for by sham-operated groups. Alternative explanations, such as neuroprotective effects of altered glial gene expression, modulation of neuroinflammation, or loss of neurotoxic glial subpopulations following PTBP1 knockdown (McDowall et al., 2024), deserve further investigation but remain speculative.

### Validation strategies

**FIGURE 2:**
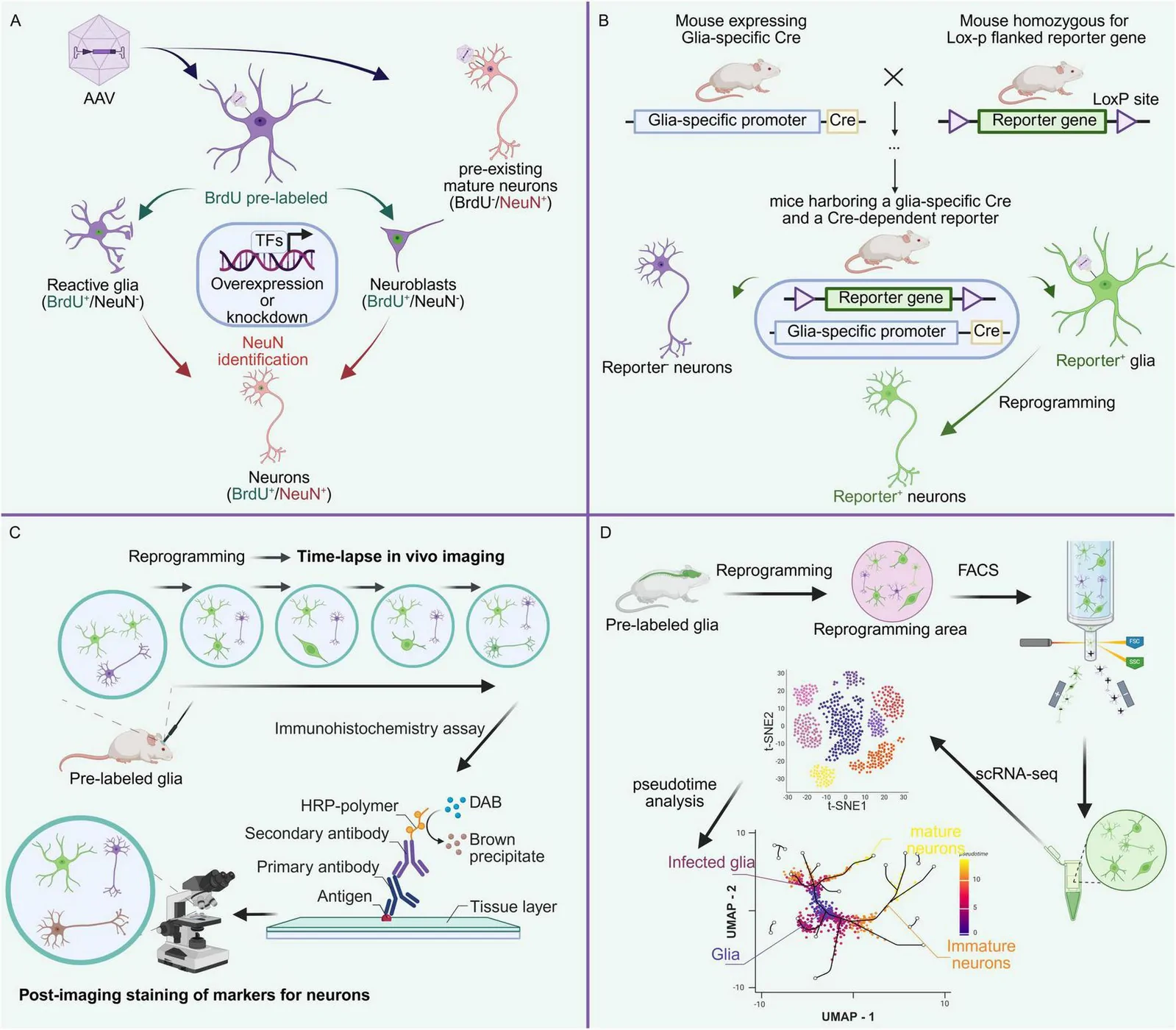
Strategies to minimize false positive results due to AAV leakage. Here are four methods to confirm neuronal identity and track the reprogramming process, minimizing false positives resulting from AAV leakage. **(A)** BrdU/EdU incorporation labels proliferating glia or reprogramming-derived neuroblasts, with neuronal identity confirmed through NeuN/MAP2 staining. **(B)** Genetic lineage tracing is performed using glia-specific Cre and Cre-dependent reporter mice, with reporter-positive neurons identified by immunohistochemistry. **(C)** Time-lapse *in vivo* imaging tracks labeled glia after viral induction, followed by immunostaining to confirm neuronal conversion. **(D)** Single-cell RNA sequencing of reporter-positive glia, sorted by fluorescence-activated cell sorting, enables trajectory analysis from glia to immature and mature neurons. FACS, fluorescence-activated cell sorting. Created in BioRender. Chen, Y. (2026) https://BioRender.com/8sh95d8.

The lessons from the PTBP1 controversy necessitate a rigorous validation framework for all future *in vivo* direct neuronal reprogramming studies. We propose the following essential verification strategies (Figure 2).

First, proliferation-based cell tracking using BrdU or EdU incorporation should be employed to pre-label dividing glial cells prior to reprogramming induction. Subsequent co-localization of BrdU/EdU with mature neuronal markers including NeuN and MAP2 in the converted cells provides evidence that these neurons originated from proliferative glial populations rather than from pre-existing post-mitotic neurons. Second, genetic lineage tracing using the Cre-loxP system, driven by glial-specific promoters (e.g., GFAP-Cre, Aldh1l1-CreERT2), should be considered the gold standard for confirming the glial origin of reprogrammed neurons. Importantly, the fidelity of the Cre driver itself must be validated, as leaky Cre expression in neurons or instability caused by downstream gene sequences can compromise lineage-tracing conclusions (Wang and Zhang, 2022). Third, *in vivo* time-lapse imaging technologies, such as two-photon microscopy with chronic cranial windows, can enable real-time monitoring of the morphological and molecular transitions during reprogramming, providing direct visual evidence of cell fate conversion. Fourth, single-cell RNA sequencing of cells within the reprogramming area offers an unbiased approach to assess whether transcriptomic trajectories consistent with a glial-to-neuronal transition are present. The identification of intermediate cell states along a continuous pseudotime trajectory from glial to neuronal identity would provide strong corroborative evidence for genuine reprogramming. Additionally, reprogramming experiments should proactively avoid regions rich in endogenous neural stem cells to prevent confounding neurogenesis from resident progenitors.

### Future perspectives

Collectively, this body of evidence suggests that the efficiency and specificity of AAV-mediated *in vivo* glial-to-neuron conversion, at least for NeuroD1 and PTBP1-based approaches, may be substantially lower than initially reported. However, it is essential to recognize that these controversies pertain specifically to *in vivo* AAV-mediated paradigms and do not invalidate the well-controlled *in vitro* reprogramming evidence using these same factors, where environmental variables are tightly regulated and lineage contamination is not a concern. The *in vitro* foundation remains robust and continues to offer valuable insights into the molecular mechanisms governing cell fate conversion. The challenge, therefore, lies in developing strategies that can faithfully translate these *in vitro* capabilities into the *in vivo* setting. Promising alternatives, such as hydrogel-mediated sustained delivery, astrocyte-targeted nanoparticle formulations, and non-viral delivery systems, are being explored to overcome the limitations of direct intracranial AAV injection (McCaughey-Chapman et al., 2024; McDowall et al., 2024). Furthermore, even if complete glial-to-neuron conversion proves difficult to achieve *in vivo*, partial reprogramming intermediates which often lead to cell death may be therapeutically exploitable, particularly in the context of glioblastoma therapy where inducing tumor cell death is the desired outcome.

## Discussion

Direct neuronal reprogramming has advanced rapidly across multiple neurological conditions, but the PTBP1 controversy and the associated AAV leakage artifacts demonstrate that clinical translation will be determined not by enthusiasm alone, but by mechanistic precision, faithful preclinical modeling, and translational realism.

For any clinical application, ensuring the long-term survival and functional integration of newly reprogrammed neurons within the pathological central nervous system remains a formidable obstacle. Chronic neuroinflammation, reactive gliosis, and metabolic dysregulation create a non-permissive milieu in which induced neurons often fail to mature or persist, yet early reprogramming studies focused predominantly on the cell-autonomous action of reprogramming factors, largely overlooking the surrounding tissue microenvironment. Recent work has begun to address this gap. Promoting anti-inflammatory M2 microglial polarization through the IL-3/IL-3Rα axis enhances clearance of toxic protein aggregates while reducing cytotoxicity against newly reprogrammed neurons (Ma et al., 2024; McAlpine et al., 2021), and targeting immunometabolic pathways such as lactate metabolism and adenosine signaling mitigates chronic neuroinflammation (Lepiarz-Raba et al., 2023; Wang Q. et al., 2022; Wang H. et al., 2024). TREM2-activating antibody transport vehicles further demonstrate the feasibility of restoring brain glucose metabolism and enhancing microglia-mediated trophic support (van Lengerich et al., 2023), while engineered chemotactic gradients such as CXCL12/CXCR4 signaling could guide reprogrammed neurons toward specific lesion sites (Bajetto et al., 2001; Xu H. et al., 2023). Yet substantial obstacles remain: the dynamic interactions between immune cells and induced neurons are difficult to recapitulate in two-dimensional cultures, and the combinatorial complexity of integrating immunomodulators with reprogramming factors quickly exceeds the throughput of traditional preclinical platforms.

These limitations point to a deeper need for physiologically faithful and scalable platforms in which to dissect neuro-immune crosstalk and screen combinatorial interventions. Immune-competent neural organoids incorporating microglia have begun to decode complex neuro-immune interactions that two-dimensional cultures cannot capture, refining the identification of reprogramming targets and conditions (Mrza et al., 2024). Assembloids, generated by combining region-specific organoids, further enable the reconstruction of interregional neuronal migration and circuit connectivity, as demonstrated by a four-part human sensory assembloid that reproduced polysynaptic signal transmission (Kim J. I. et al., 2025). Patient-derived forebrain assembloids have also recapitulated Timothy syndrome phenotypes and enabled preclinical evaluation of a corrective antisense oligonucleotide, highlighting their potential for disease modeling and therapeutic development (Chen et al., 2024). Region-specific organoids derived from directly reprogrammed cells have also demonstrated therapeutic potential, as exemplified by the conversion of human astrocytes into spinal cord organoids through OCT4 overexpression combined with a defined small-molecule cocktail, which upon transplantation into complete spinal cord injury models achieved synaptic integration with host neurons (Xu H. et al., 2023). For scalable neuronal culture, automated systems integrating programmable medium exchange, environmental monitoring, imaging, and electrophysiological recording could reduce operator-dependent variability and enable continuous, feedback-controlled maintenance (Voitiuk et al., 2025). Patterned microwells and spinner-flask bioreactors can further standardize aggregate size and support large-scale culture; when combined with automated high-content imaging, these systems have enabled the reproducible production of thousands of brain organoids and medium-throughput drug screening (Ramani et al., 2024). Organ-on-a-chip platforms extend these capabilities by enabling precise control of chemokine gradients and dynamic fluid environments (Holloway et al., 2021; Sin et al., 2004), and when patient-derived organoids are coupled with multiplexed microfluidic chips, they support high-throughput screening of reprogramming factor and immunomodulator combinations alongside real-time tracking of neuro-immune biomarkers (Yang et al., 2024). A fundamental limitation persists, however: precise spatiotemporally programmable control over organoid patterning and gene expression remains technically challenging, resulting in inconsistent heterogeneity and limited reproducibility. This bottleneck is particularly problematic for studies aiming to generate specific neuronal subtypes, since faithful specification depends on reproducing precisely timed developmental signals.

Achieving this level of precision, both within organoid platforms and ultimately in clinical applications, demands tools beyond conventional viral or genetic delivery. Different neurological diseases require fundamentally different neuronal populations, and faithful subtype specification therefore depends on reproducing developmental sequences with precision conventional methods cannot achieve. Optogenetic control of neurogenic transcription factor expression, such as light-inducible activation of Ascl1 or NeuroD1, enables sequential factor activation patterns that mimic natural developmental programs while minimizing off-target effects (Legnini et al., 2023; Shiri et al., 2019). The combination of optogenetics with CRISPR-based RNA perturbation has demonstrated spatiotemporal control of gene expression in human organoids, with localized activation of Sonic Hedgehog signaling driving patterned neurodevelopment and directly addressing the patterning bottleneck described above (Legnini et al., 2023). Spatial transcriptomics complements this approach by identifying subtype-specific molecular signatures within native brain tissue, informing the selection and timing of reprogramming factors tailored to specific subtypes and brain regions (Wang G. et al., 2024). Building on these high-dimensional datasets, artificial intelligence and machine-learning models could prioritize reprogramming-factor combinations, predict cell-state transitions, and optimize induction protocols (Li et al., 2025; Wytock and Motter, 2024). Deep-learning analysis of cell-culture images may further automate the assessment of conversion efficiency, neuronal maturation, and batch quality, while integration with patient-specific molecular and clinical data could support efficacy and safety prediction in translational studies (Zhu et al., 2021). Optogenetic studies in disease models have also revealed novel circuit-level mechanisms such as the role of medial mammillary body dysfunction in early Alzheimer’s disease (Huang et al., 2025) and midbrain dopaminergic neuron stimulation in restoring hippocampal plasticity (Ficchì et al., 2025), that identify new therapeutic targets amenable to reprogramming-based interventions. Despite this progress, optogenetic approaches face their own translational challenges: prolonged light delivery can cause heating, tissue damage, and unintended transcriptional activation (Cheng et al., 2016; Tyssowski and Gray, 2019), sustained opsin expression may lead to dark activity and cellular toxicity, and the invasiveness of current fiber-optic delivery systems restricts both behavioral studies and clinical applicability. Sex-dependent differences such as elevated bFGF levels in females have further been reported to inhibit the therapeutic effects of optogenetic astrocyte activation in post-stroke brain repair (Shao et al., 2025).

Beyond these technical bottlenecks, several fundamental questions remain unresolved. The extent to which induced neurons are functionally and molecularly equivalent to their endogenous counterparts is still unclear; although directly reprogrammed neurons retain donor-specific aging signatures advantageous for disease modeling (Mertens et al., 2015, 2018), it remains uncertain whether they replicate the precise electrophysiological, synaptic, and metabolic properties required for stable circuit integration *in vivo*. Equally, the long-term consequences of epigenetic memory carried over from the cell of origin are poorly understood, as residual epigenetic marks may compromise neuronal maturation or predispose induced neurons to dysfunction under disease-relevant stress (Mertens et al., 2021; Traxler et al., 2021). The therapeutic value of incomplete or partial reprogramming has likewise been largely overlooked, even though intermediate-state cell death could be deliberately exploited in oncological contexts such as glioblastoma, where induction of tumor cell death is itself the therapeutic objective.

**FIGURE 3:**
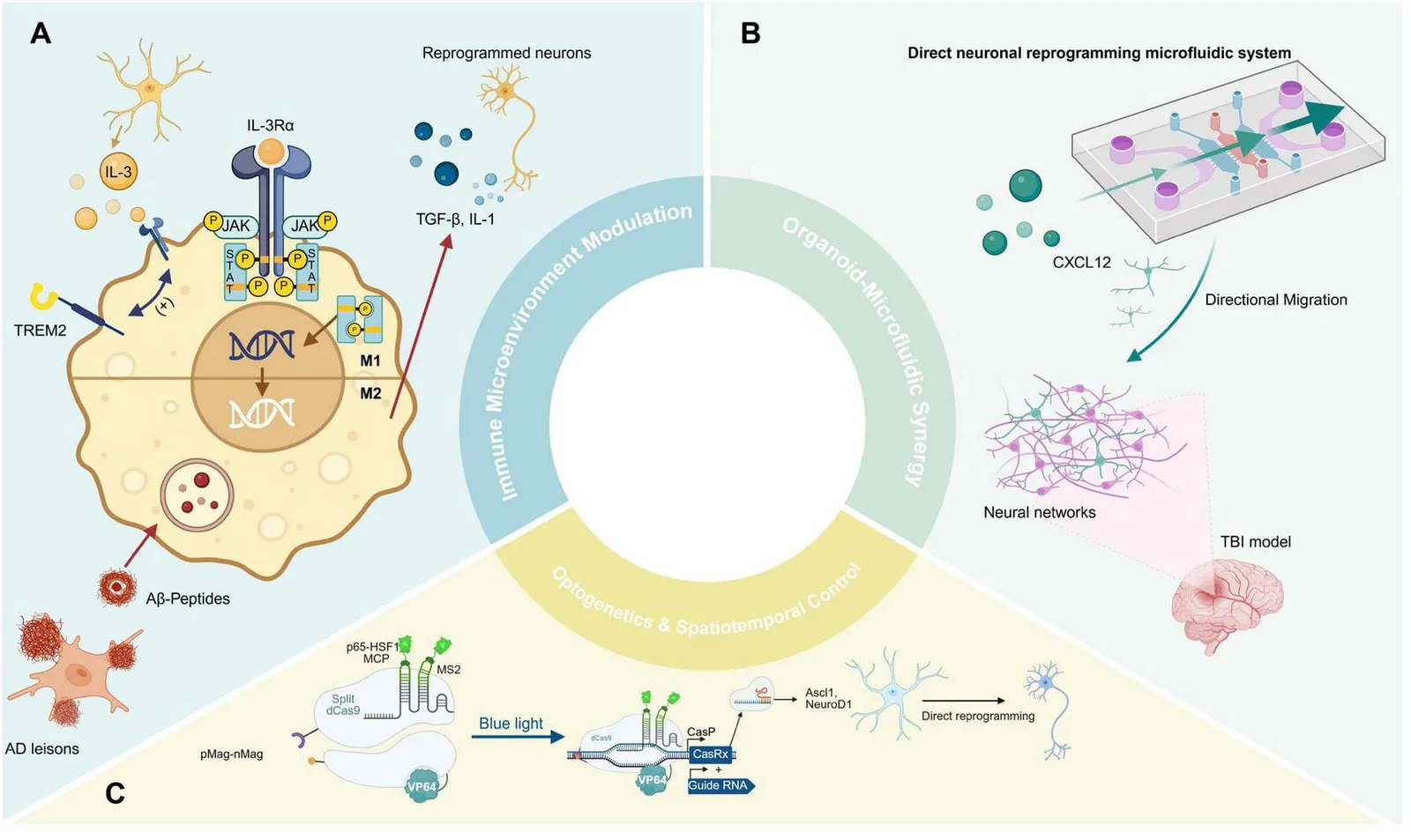
Future directions to advance direct neuronal reprogramming toward clinical application. This schematic illustrates three existing examples of future directions for improving direct neuronal reprogramming technologies. (A) Immune microenvironment modulation: promoting M2 macrophage polarization and cytokine regulation to clear pathological proteins and protect the neuronal regeneration environment. (B) Organoid-microfluidic synergy: integrating organoid culture with microfluidic platforms enables the direct migration of reprogrammed neurons, which can be guided by a CXCL12 concentration gradient in a traumatic brain injury (TBI) model. (C) Optogenetic and spatiotemporal control: light-inducible CRISPR systems allow precise regulation of reprogramming factors (e.g., Ascl1, NeuroD1), enhancing efficiency, neuronal subtype specification, and functional integration. AD, Alzheimer’s disease; Aβ-Peptides: Amyloid-beta peptides; TBI: traumatic brain injury. Created in BioRender. Chen, Y. (2026) https://BioRender.com/325s6gk.

Taken together, the controversies surrounding *in vivo* reprogramming have paradoxically strengthened the field by exposing the gap between reporter-based observations and genuine cell fate conversion. The next stage of direct neuronal reprogramming should therefore be defined not by additional proof-of-concept demonstrations alone, but by rigorous lineage tracing, functional validation, and disease-specific translational prioritization. Realizing this clinical promise will require sustained convergence across immune modulation, physiologically faithful disease modeling, spatially resolved molecular profiling, precision gene regulation, and long-term safety assessment (Figure 3).

## References

[B1] Abernathy D. G. Kim W. K. McCoy M. J. Lake A. M. Ouwenga R. Lee S. W. et al. (2017). MicroRNAs induce a permissive chromatin environment that enables neuronal subtype-specific reprogramming of adult human fibroblasts. *Cell Stem Cell* 21 332–48.e9. 10.1016/j.stem.2017.08.002 28886366 PMC5679239

[B2] Ambasudhan R. Talantova M. Coleman R. Yuan X. Zhu S. Lipton S. A. et al. (2011). Direct reprogramming of adult human fibroblasts to functional neurons under defined conditions. *Cell Stem Cell* 9 113–118. 10.1016/j.stem.2011.07.002 21802386 PMC4567246

[B3] Bajetto A. Bonavia R. Barbero S. Florio T. Schettini G. (2001). Chemokines and their receptors in the central nervous system. *Front. Neuroendocrinol.* 22 147–184. 10.1006/frne.2001.0214 11456467

[B4] Ballas N. Grunseich C. Lu D. D. Speh J. C. Mandel G. (2005). REST and its corepressors mediate plasticity of neuronal gene chromatin throughout neurogenesis. *Cell* 121 645–657. 10.1016/j.cell.2005.03.013 15907476

[B5] Bauer P. O. Dunmore J. H. Sasaguri H. Matoska V. (2019). Neurons induced from fibroblasts of c9ALS/FTD patients reproduce the pathology seen in the central nervous system. *Front. Neurosci.* 13:935. 10.3389/fnins.2019.00935 31551693 PMC6743368

[B6] Belair-Hickey J. J. Fahmy A. Zhang W. Sajid R. S. Coles B. L. K. Salter M. W. et al. (2024). Neural crest precursors from the skin are the primary source of directly reprogrammed neurons. *Stem Cell Rep.* 19 1620–1634. 10.1016/j.stemcr.2024.10.003 39486406 PMC11589197

[B7] Bocchi R. Masserdotti G. Götz M. (2022). Direct neuronal reprogramming: Fast forward from new concepts toward therapeutic approaches. *Neuron* 110 366–393. 10.1016/j.neuron.2021.11.023 34921778

[B8] Boudreau-Pinsonneault C. David L. A. Lourenço Fernandes J. A. Javed A. Fries M. Mattar P. et al. (2023). Direct neuronal reprogramming by temporal identity factors. *Proc. Natl. Acad. Sci. U. S. A.* 120:e2122168120. 10.1073/pnas.2122168120 37126716 PMC10175841

[B9] Burns T. C. Quinones-Hinojosa A. (2021). Regenerative medicine for neurological diseases-will regenerative neurosurgery deliver? *BMJ* 373:n955. 10.1136/bmj.n955 34162530

[B10] Cates K. Yuan L. Yang Y. Yoo A. S. (2025). Fate erasure logic of gene networks underlying direct neuronal conversion of somatic cells by microRNAs. *Cell Rep.* 44:115153. 10.1016/j.celrep.2024.115153 39756035 PMC11834941

[B11] Chanda S. Ang C. E. Davila J. Pak C. Mall M. Lee Q. Y. et al. (2014). Generation of induced neuronal cells by the single reprogramming factor ASCL1. *Stem Cell Rep.* 3 282–296. 10.1016/j.stemcr.2014.05.020 25254342 PMC4176533

[B12] Chanda S. Marro S. Wernig M. Südhof T. C. (2013). Neurons generated by direct conversion of fibroblasts reproduce synaptic phenotype caused by autism-associated neuroligin-3 mutation. *Proc. Natl. Acad. Sci. U. S. A.* 110 16622–16627. 10.1073/pnas.1316240110 24046374 PMC3799342

[B13] Chang J. H. Tsai P. H. Wang K. Y. Wei Y. T. Chiou S. H. Mou C. Y. (2018). Generation of functional dopaminergic neurons from reprogramming fibroblasts by nonviral-based mesoporous silica nanoparticles. *Sci. Rep.* 8:11. 10.1038/s41598-017-18324-8 29311646 PMC5758610

[B14] Chen W. Zheng Q. Huang Q. Ma S. Li M. (2022). Repressing PTBP1 fails to convert reactive astrocytes to dopaminergic neurons in a 6-hydroxydopamine mouse model of Parkinson’s disease. *Elife* 11:e75636. 10.7554/eLife.75636 35535997 PMC9208759

[B15] Chen X. Birey F. Li M. Y. Revah O. Levy R. Thete M. V. et al. (2024). Antisense oligonucleotide therapeutic approach for Timothy syndrome. *Nature* 628 818–825. 10.1038/s41586-024-07310-6 38658687 PMC11043036

[B16] Chen Y. C. Ma N. X. Pei Z. F. Wu Z. Do-Monte F. H. Keefe S. et al. (2020). A NeuroD1 AAV-Based gene therapy for functional brain repair after ischemic injury through In Vivo astrocyte-to-neuron conversion. *Mol. Ther.* 28 217–234. 10.1016/j.ymthe.2019.09.003 31551137 PMC6952185

[B17] Cheng K. P. Kiernan E. A. Eliceiri K. W. Williams J. C. Watters J. J. (2016). Blue light modulates murine microglial gene expression in the absence of optogenetic protein expression. *Sci. Rep.* 6:21172. 10.1038/srep21172 26883795 PMC4756664

[B18] Cheng X. Tan Z. Huang X. Yuan Y. Qin S. Gu Y. et al. (2019). Inhibition of glioma development by ASCL1-mediated direct neuronal reprogramming. *Cells* 8:571. 10.3390/cells8060571 31212628 PMC6627512

[B19] Colasante G. Rubio A. Massimino L. Broccoli V. (2019). Direct neuronal reprogramming reveals unknown functions for known transcription factors. *Front. Neurosci.* 13:283. 10.3389/fnins.2019.00283 30971887 PMC6445133

[B20] Connor B. Firmin E. McCaughey-Chapman A. Monk R. Lee K. Liot S. et al. (2018). Conversion of adult human fibroblasts into neural precursor cells using chemically modified mRNA. *Heliyon* 4:e00918. 10.1016/j.heliyon.2018.e00918 30450440 PMC6226601

[B21] Dai S. Wang C. Zhang C. Feng L. Zhang W. Zhou X. et al. (2022). PTB: Not just a polypyrimidine tract-binding protein. *J. Cell Physiol* 237 2357–2373. 10.1002/jcp.30716 35288937

[B22] Davis R. L. Weintraub H. Lassar A. B. (1987). Expression of a single transfected cDNA converts fibroblasts to myoblasts. *Cell* 51 987–1000. 10.1016/0092-8674(87)90585-x 3690668

[B23] Ebrahimi A. Keske E. Mehdipour A. Ebrahimi-Kalan A. Ghorbani M. (2019). Somatic cell reprogramming as a tool for neurodegenerative diseases. *Biomed. Pharmacother.* 112:108663. 10.1016/j.biopha.2019.108663 30970509

[B24] Feigin V. L. Vos T. Alahdab F. Amit A. M. L. Bärnighausen T. W. Beghi E. et al. (2021). Burden of neurological disorders across the us from 1990-2017: A global burden of disease study. *JAMA Neurol.* 78 165–176. 10.1001/jamaneurol.2020.4152 33136137 PMC7607495

[B25] Feigin V. L. Vos T. Nichols E. Owolabi M. O. Carroll W. M. Dichgans M. et al. (2020). The global burden of neurological disorders: Translating evidence into policy. *Lancet Neurol.* 19 255–265. 10.1016/s1474-4422(19)30411-9 31813850 PMC9945815

[B26] Feng S. Zhang T. Ke W. Xiao Y. Guo Z. Lu C. et al. (2022). The long-term survival and functional maturation of human iNPC-derived neurons in the basal forebrain of cynomolgus monkeys. *Life Med.* 1 196–206. 10.1093/lifemedi/lnac008 39871935 PMC11749281

[B27] Fermi V. Warta R. Wöllner A. Lotsch C. Jassowicz L. Rapp C. et al. (2023). Effective reprogramming of patient-derived M2-Polarized glioblastoma-associated microglia/macrophages by treatment with GW2580. *Clin. Cancer Res.* 29 4685–4697. 10.1158/1078-0432.Ccr-23-0576 37682326

[B28] Ficchì S. Cauzzi E. La Barbera L. De Paolis M. L. Loffredo G. Spoleti E. et al. (2025). Optogenetic stimulation of midbrain dopaminergic neurons rescues hippocampal synaptic plasticity deficits in a mouse model of Alzheimer’s disease. *Transl. Psychiatry* 15:371. 10.1038/s41398-025-03588-w 41053038 PMC12500900

[B29] Gage F. H. (2000). Mammalian neural stem cells. *Science* 287 1433–1438. 10.1126/science.287.5457.1433 10688783

[B30] Gallego-Perez D. Otero J. J. Czeisler C. Ma J. Ortiz C. Gygli P. et al. (2016). Deterministic transfection drives efficient nonviral reprogramming and uncovers reprogramming barriers. *Nanomedicine* 12 399–409. 10.1016/j.nano.2015.11.015 26711960 PMC5161095

[B31] Giacomoni J. Laurin K. Parmar M. Habekost M. (2025). Protocol for 3D direct reprogramming of human glia into dopaminergic neurons with gene expression and immunocytochemistry validation. *STAR Protoc.* 6:104088. 10.1016/j.xpro.2025.104088 40946308 PMC12766404

[B32] Giehrl-Schwab J. Giesert F. Rauser B. Lao C. L. Hembach S. Lefort S. et al. (2022). Parkinson’s disease motor symptoms rescue by CRISPRa-reprogramming astrocytes into GABAergic neurons. *EMBO Mol. Med.* 14:e14797. 10.15252/emmm.202114797 35373464 PMC9081909

[B33] Golpour M. Prsian H. (2025). From Pluripotency to Patients: Making Induced pluripotent stem cell (iPSC) Therapies Safer. *Int. J. Mol. Cell Med.* 14 976–979. 10.22088/ijmcm.Bums.14.4.976 42016034 PMC13092833

[B34] Gong L. Yan Q. Zhang Y. Fang X. Liu B. Guan X. (2019). Cancer cell reprogramming: A promising therapy converting malignancy to benignity. *Cancer Commun.* 39:48. 10.1186/s40880-019-0393-5 31464654 PMC6716904

[B35] Gooch C. L. Pracht E. Borenstein A. R. (2017). The burden of neurological disease in the United States: A summary report and call to action. *Ann. Neurol.* 81 479–484. 10.1002/ana.24897 28198092

[B36] Guo Z. Zhang L. Wu Z. Chen Y. Wang F. Chen G. (2014). In vivo direct reprogramming of reactive glial cells into functional neurons after brain injury and in an Alzheimer’s disease model. *Cell Stem Cell* 14 188–202. 10.1016/j.stem.2013.12.001 24360883 PMC3967760

[B37] Heinrich C. Blum R. Gascón S. Masserdotti G. Tripathi P. Sánchez R. et al. (2010). Directing astroglia from the cerebral cortex into subtype specific functional neurons. *PLoS Biol.* 8:e1000373. 10.1371/journal.pbio.1000373 20502524 PMC2872647

[B38] Herdy J. R. Traxler L. Agarwal R. K. Karbacher L. Schlachetzki J. C. M. Boehnke L. et al. (2022). Increased post-mitotic senescence in aged human neurons is a pathological feature of Alzheimer’s disease. *Cell Stem Cell* 29 1637–52.e6. 10.1016/j.stem.2022.11.010 36459967 PMC10093780

[B39] Hoang T. Kim D. W. Appel H. Pannullo N. A. Leavey P. Ozawa M. et al. (2022). Genetic loss of function of Ptbp1 does not induce glia-to-neuron conversion in retina. *Cell Rep.* 39:110849. 10.1016/j.celrep.2022.110849 35705053 PMC9619396

[B40] Hoang T. Wang J. Boyd P. Wang F. Santiago C. Jiang L. et al. (2020). Gene regulatory networks controlling vertebrate retinal regeneration. *Science* 370:eabb8598. 10.1126/science.abb8598 33004674 PMC7899183

[B41] Holloway P. M. Willaime-Morawek S. Siow R. Barber M. Owens R. M. Sharma A. D. et al. (2021). Advances in microfluidic in vitro systems for neurological disease modeling. *J. Neurosci. Res.* 99 1276–1307. 10.1002/jnr.24794 33583054

[B42] Hu W. Qiu B. Guan W. Wang Q. Wang M. Li W. et al. (2015). Direct conversion of normal and Alzheimer’s disease human fibroblasts into neuronal cells by small molecules. *Cell Stem Cell* 17 204–212. 10.1016/j.stem.2015.07.006 26253202

[B43] Huang D. Long Y. Yang Y. Wu J. Yang J. Huang Y. et al. (2025). Impairments of spatial memory retrieval via medial mammillary body dysfunction in Alzheimer’s disease model. *Transl. Psychiatry* 15:353. 10.1038/s41398-025-03583-1 41053035 PMC12501326

[B44] Ieda M. Fu J. D. Delgado-Olguin P. Vedantham V. Hayashi Y. Bruneau B. G. et al. (2010). Direct reprogramming of fibroblasts into functional cardiomyocytes by defined factors. *Cell* 142 375–386. 10.1016/j.cell.2010.07.002 20691899 PMC2919844

[B45] Jiang M. Q. Yu S. P. Estaba T. Choi E. Berglund K. Gu X. et al. (2024). Reprogramming glioblastoma cells into non-cancerous neuronal cells as a novel anti-cancer strategy. *Cells* 13:897. 10.3390/cells13110897 38891029 PMC11171681

[B46] Jorstad N. L. Wilken M. S. Grimes W. N. Wohl S. G. VandenBosch L. S. Yoshimatsu T. et al. (2017). Stimulation of functional neuronal regeneration from Müller glia in adult mice. *Nature* 548 103–107. 10.1038/nature23283 28746305 PMC5991837

[B47] Kajtez J. Laurin K. Nilsson F. Bruzelius A. Cepeda-Prado E. Birtele M. et al. (2025). Three-dimensional cell-cell interactions promote direct reprogramming of patient fibroblasts into functional and transplantable neurons. *Sci. Adv.* 11:eadq7855. 10.1126/sciadv.adq7855 40479059 PMC12143395

[B48] Karaosmanoglu B. Imren G. Ozisin M. S. Reçber T. Simsek Kiper P. O. Haliloglu G. et al. (2024). Ex vivo disease modelling of Rett syndrome: The transcriptomic and metabolomic implications of direct neuronal conversion. *Mol. Biol. Rep.* 51:979. 10.1007/s11033-024-09915-6 39269588

[B49] Kim J. I. Imaizumi K. Jurjuţ O. Kelley K. W. Wang D. Thete M. V. et al. (2025). Human assembloid model of the ascending neural sensory pathway. *Nature* 642 143–153. 10.1038/s41586-025-08808-3 40205039 PMC12137141

[B50] Kim J. Kim S. Hwang Y. An S. Park J. Kwon Y. B. et al. (2025). Electromagnetized MXenes enhance the efficient direct reprogramming of dopamine neurons for Parkinson’s disease therapy. *ACS Nano* 19 16744–16759. 10.1021/acsnano.5c01457 40257388

[B51] Kraskovskaya N. Bolshakova A. Khotin M. Bezprozvanny I. Mikhailova N. (2023). Protocol optimization for direct reprogramming of primary human fibroblast into induced striatal neurons. *Int. J. Mol. Sci.* 24:6799. 10.3390/ijms24076799 37047770 PMC10095147

[B52] Legnini I. Emmenegger L. Zappulo A. Rybak-Wolf A. Wurmus R. Martinez A. O. et al. (2023). Spatiotemporal, optogenetic control of gene expression in organoids. *Nat. Methods* 20 1544–1552. 10.1038/s41592-023-01986-w 37735569 PMC10555836

[B53] Lepiarz-Raba I. Gbadamosi I. Florea R. Paolicelli R. C. Jawaid A. (2023). Metabolic regulation of microglial phagocytosis: Implications for Alzheimer’s disease therapeutics. *Transl. Neurodegener.* 12:48. 10.1186/s40035-023-00382-w 37908010 PMC10617244

[B54] Li C. Chen S. Chen Y. Bian H. Hao M. Wei L. et al. (2025). TFcomb identifies transcription factor combinations for cellular reprogramming based on single-cell multiomics data. *Genome Res.* 35 1429–1439. 10.1101/gr.279955.124 40210438 PMC12129019

[B55] Li S. Shi Y. Yao X. Wang X. Shen L. Rao Z. et al. (2019). Conversion of astrocytes and fibroblasts into functional noradrenergic neurons. *Cell Rep.* 28 682–97.e7. 10.1016/j.celrep.2019.06.042 31315047

[B56] Li X. Zuo X. Jing J. Ma Y. Wang J. Liu D. et al. (2015). Small-Molecule-Driven direct reprogramming of mouse fibroblasts into functional neurons. *Cell Stem Cell* 17 195–203. 10.1016/j.stem.2015.06.003 26253201

[B57] Liou R. H. Edwards T. L. Martin K. R. Wong R. C. (2020). Neuronal reprogramming for tissue repair and neuroregeneration. *Int. J. Mol. Sci.* 21:4273. 10.3390/ijms21124273 32560072 PMC7352898

[B58] Liu H. Ma Y. Gao N. Zhou Y. Li G. Zhu Q. et al. (2025). Identification and characterization of human retinal stem cells capable of retinal regeneration. *Sci. Transl. Med.* 17:ead6864. 10.1126/scitranslmed.adp6864 40138453

[B59] Liu J. Xin X. Sun J. Fan Y. Zhou X. Gong W. et al. (2024). Dual-targeting AAV9P1-mediated neuronal reprogramming in a mouse model of traumatic brain injury. *Neural Regen. Res.* 19 629–635. 10.4103/1673-5374.380907 37721294 PMC10581548

[B60] Liu M. L. Zang T. Zhang C. L. (2016). Direct lineage reprogramming reveals disease-specific phenotypes of motor neurons from human ALS patients. *Cell Rep.* 14 115–128. 10.1016/j.celrep.2015.12.018 26725112 PMC4706770

[B61] Liu T. Jin D. Le S. B. Chen D. Sebastian M. Riva A. et al. (2024). Machine learning-directed conversion of glioblastoma cells to dendritic cell-like antigen-presenting cells as cancer immunotherapy. *Cancer Immunol. Res.* 12 1340–1360. 10.1158/2326-6066.Cir-23-0721 39051633 PMC11491168

[B62] Liu Y. Xue Y. Ridley S. Zhang D. Rezvani K. Fu X. D. et al. (2014). Direct reprogramming of Huntington’s disease patient fibroblasts into neuron-like cells leads to abnormal neurite outgrowth, increased cell death, and aggregate formation. *PLoS One* 9:e109621. 10.1371/journal.pone.0109621 25275533 PMC4183653

[B63] Lu Y. Brommer B. Tian X. Krishnan A. Meer M. Wang C. et al. (2020). Reprogramming to recover youthful epigenetic information and restore vision. *Nature* 588 124–129. 10.1038/s41586-020-2975-4 33268865 PMC7752134

[B64] Ma R. Mu Q. Xi Y. Liu G. Liu C. (2024). Nanotechnology for tau pathology in Alzheimer’s disease. *Mater. Today Bio.* 27:101145. 10.1016/j.mtbio.2024.101145 39070098 PMC11283088

[B65] Marcucci F. Murcia-Belmonte V. Wang Q. Coca Y. Ferreiro-Galve S. Kuwajima T. et al. (2016). The ciliary margin zone of the mammalian retina generates retinal ganglion cells. *Cell Rep.* 17 3153–3164. 10.1016/j.celrep.2016.11.016 28009286 PMC5234854

[B66] McAlpine C. S. Park J. Griciuc A. Kim E. Choi S. H. Iwamoto Y. et al. (2021). Astrocytic interleukin-3 programs microglia and limits Alzheimer’s disease. *Nature* 595 701–706. 10.1038/s41586-021-03734-6 34262178 PMC8934148

[B67] McCaughey-Chapman A. Burgers A. L. Combrinck C. Marriott L. Gordon D. Connor B. (2024). Reprogrammed human lateral ganglionic eminence precursors generate striatal neurons and restore motor function in a rat model of Huntington’s disease. *Stem Cell Res. Ther.* 15:448. 10.1186/s13287-024-04057-9 39578834 PMC11583420

[B68] McDowall S. Bagda V. Hodgetts S. Mastaglia F. Li D. (2024). Controversies and insights into PTBP1-related astrocyte-neuron transdifferentiation: Neuronal regeneration strategies for Parkinson’s and Alzheimer’s disease. *Transl. Neurodegener.* 13:59. 10.1186/s40035-024-00450-9 39627843 PMC11613593

[B69] Mertens J. Herdy J. R. Traxler L. Schafer S. T. Schlachetzki J. C. M. Böhnke L. et al. (2021). Age-dependent instability of mature neuronal fate in induced neurons from Alzheimer’s patients. *Cell Stem Cell* 28 1533–48.e6. 10.1016/j.stem.2021.04.004 33910058 PMC8423435

[B70] Mertens J. Paquola A. C. M. Ku M. Hatch E. Böhnke L. Ladjevardi S. et al. (2015). Directly reprogrammed human neurons retain aging-associated transcriptomic signatures and reveal age-related nucleocytoplasmic defects. *Cell Stem Cell* 17 705–718. 10.1016/j.stem.2015.09.001 26456686 PMC5929130

[B71] Mertens J. Reid D. Lau S. Kim Y. Gage F. H. (2018). Aging in a dish: IPSC-Derived and directly induced neurons for studying brain aging and age-related neurodegenerative diseases. *Annu. Rev. Genet.* 52 271–293. 10.1146/annurev-genet-120417-031534 30208291 PMC6415910

[B72] Monk R. Lee K. Jones K. S. Connor B. (2021). Directly reprogrammed Huntington’s disease neural precursor cells generate striatal neurons exhibiting aggregates and impaired neuronal maturation. *Stem Cells* 39 1410–1422. 10.1002/stem.3420 34028139

[B73] Monzón-Casanova E. Matheson L. S. Tabbada K. Zarnack K. Smith C. W. Turner M. (2020). Polypyrimidine tract-binding proteins are essential for B cell development. *Elife* 9:e53557. 10.7554/eLife.53557 32081131 PMC7058386

[B74] Moskowitz M. A. Lo E. H. Iadecola C. (2010). The science of stroke: Mechanisms in search of treatments. *Neuron* 67 181–198. 10.1016/j.neuron.2010.07.002 20670828 PMC2957363

[B75] Mrza M. A. He J. Wang Y. (2024). Integration of iPSC-Derived microglia into brain organoids for neurological research. *Int. J. Mol. Sci.* 25:3148. 10.3390/ijms25063148 38542121 PMC10970489

[B76] Nagai H. Saito M. Iwata H. (2024). Direct conversion of urine-derived cells into functional motor neuron-like cells by defined transcription factors. *Sci. Rep.* 14:27011. 10.1038/s41598-024-73759-0 39505927 PMC11541886

[B77] Oh Y. M. Lee S. W. Kim W. K. Chen S. Church V. A. Cates K. et al. (2022). Age-related Huntington’s disease progression modeled in directly reprogrammed patient-derived striatal neurons highlights impaired autophagy. *Nat. Neurosci.* 25 1420–1433. 10.1038/s41593-022-01185-4 36303071 PMC10162007

[B78] Pfisterer U. Ek F. Lang S. Soneji S. Olsson R. Parmar M. (2016). Small molecules increase direct neural conversion of human fibroblasts. *Sci. Rep.* 6:38290. 10.1038/srep38290 27917895 PMC5137010

[B79] Poewe W. Seppi K. Tanner C. M. Halliday G. M. Brundin P. Volkmann J. et al. (2017). Parkinson disease. *Nat. Rev. Dis. Primers* 3:17013. 10.1038/nrdp.2017.13 28332488

[B80] Portela-Lomba M. Simón D. Callejo-Móstoles M. de la Fuente G. Fernández de Sevilla D. García-Escudero V. et al. (2024). Generation of functional neurons from adult human mucosal olfactory ensheathing glia by direct lineage conversion. *Cell Death Dis.* 15:478. 10.1038/s41419-024-06862-9 38961086 PMC11222439

[B81] Puglisi M. Lao C. L. Wani G. Masserdotti G. Bocchi R. Götz M. (2024). Comparing viral vectors and fate mapping approaches for astrocyte-to-neuron reprogramming in the injured mouse cerebral cortex. *Cells* 13:1408. 10.3390/cells13171408 39272980 PMC11394536

[B82] Qian H. Kang X. Hu J. Zhang D. Liang Z. Meng F. et al. (2020). Reversing a model of Parkinson’s disease with in situ converted nigral neurons. *Nature* 582 550–556. 10.1038/s41586-020-2388-4 32581380 PMC7521455

[B83] Qin H. Zhao A. Fu X. (2017). Small molecules for reprogramming and transdifferentiation. *Cell Mol. Life Sci.* 74 3553–3575. 10.1007/s00018-017-2586-x 28698932 PMC11107793

[B84] Rahman M. M. Islam M. R. Islam M. T. Harun-Or-Rashid M. Islam M. Abdullah S. et al. (2022). Stem cell transplantation therapy and neurological disorders: Current status and future perspectives. *Biology* 11:147. 10.3390/biology11010147 35053145 PMC8772847

[B85] Ramani A. Pasquini G. Gerkau N. J. Jadhav V. Vinchure O. S. Altinisik N. et al. (2024). Reliability of high-quantity human brain organoids for modeling microcephaly, glioma invasion and drug screening. *Nat. Commun.* 15:10703. 10.1038/s41467-024-55226-6 39702477 PMC11659410

[B86] Saito Y. Ishikawa M. Ohkuma M. Moody J. Mabuchi Y. Sanosaka T. et al. (2025). NEUROD1 efficiently converts peripheral blood cells into neurons with partial reprogramming by pluripotency factors. *Proc. Natl. Acad. Sci. U. S. A.* 122:e2401387122. 10.1073/pnas.2401387122 40299704 PMC12067290

[B87] Servillo F. De Carluccio M. Di Lazzaro G. Campanelli F. Marino G. Natale G. et al. (2024). Molecular and cellular determinants of L-Dopa-induced dyskinesia in Parkinson’s Disease. *NPJ Parkinsons. Dis.* 10:228. 10.1038/s41531-024-00836-6 39616186 PMC11608318

[B88] Shao X. Yao Y. Shi V. Suo Q. Wu S. Wang H. et al. (2025). bFGF-Mediated inhibition of Astrocytes’ optogenetic activation impairs neuronal repair in female rats after stroke. *Int. J. Mol. Sci.* 26 6521. 10.3390/ijms26136521 40650297 PMC12249867

[B89] Shiri Z. Simorgh S. Naderi S. Baharvand H. (2019). Optogenetics in the era of cerebral organoids. *Trends Biotechnol.* 37 1282–1294. 10.1016/j.tibtech.2019.05.009 31227305

[B90] Silber J. Lim D. A. Petritsch C. Persson A. I. Maunakea A. K. Yu M. et al. (2008). miR-124 and miR-137 inhibit proliferation of glioblastoma multiforme cells and induce differentiation of brain tumor stem cells. *BMC Med.* 6:14. 10.1186/1741-7015-6-14 18577219 PMC2443372

[B91] Sims J. R. Zimmer J. A. Evans C. D. Lu M. Ardayfio P. Sparks J. et al. (2023). Donanemab in early symptomatic Alzheimer disease: The TRAILBLAZER-ALZ 2 randomized clinical trial. *JAMA* 330 512–527. 10.1001/jama.2023.13239 37459141 PMC10352931

[B92] Sin A. Chin K. C. Jamil M. F. Kostov Y. Rao G. Shuler M. L. (2004). The design and fabrication of three-chamber microscale cell culture analog devices with integrated dissolved oxygen sensors. *Biotechnol. Prog.* 20 338–345. 10.1021/bp034077d 14763861

[B93] Soucy J. R. Aguzzi E. A. Cho J. Gilhooley M. J. Keuthan C. Luo Z. et al. (2023). Retinal ganglion cell repopulation for vision restoration in optic neuropathy: A roadmap from the RReSTORe Consortium. *Mol. Neurodegener.* 18:64. 10.1186/s13024-023-00655-y 37735444 PMC10514988

[B94] Su Z. Zang T. Liu M. L. Wang L. L. Niu W. Zhang C. L. (2014). Reprogramming the fate of human glioma cells to impede brain tumor development. *Cell Death Dis*. 5:e1463. 10.1038/cddis.2014.425 25321470 PMC4649522

[B95] Sun Z. Kwon J. S. Ren Y. Chen S. Walker C. K. Lu X. et al. (2024). Modeling late-onset Alzheimer’s disease neuropathology via direct neuronal reprogramming. *Science* 385:adl2992. 10.1126/science.adl2992 39088624 PMC11787906

[B96] Tai W. Wu W. Wang L. L. Ni H. Chen C. Yang J. et al. (2021). In vivo reprogramming of NG2 glia enables adult neurogenesis and functional recovery following spinal cord injury. *Cell Stem Cell* 28 923–37.e4. 10.1016/j.stem.2021.02.009 33675690 PMC8106641

[B97] Takahashi K. Yamanaka S. (2006). Induction of pluripotent stem cells from mouse embryonic and adult fibroblast cultures by defined factors. *Cell* 126 663–676. 10.1016/j.cell.2006.07.024 16904174

[B98] Talifu Z. Zhang C. Xu X. Pan Y. Ke H. Li Z. et al. (2024). Neuronal repair after spinal cord injury by in vivo astrocyte reprogramming mediated by the overexpression of NeuroD1 and Neurogenin-2. *Biol. Res.* 57:53. 10.1186/s40659-024-00534-w 39135103 PMC11318173

[B99] Tian Z. Guo F. Biswas S. Deng W. (2016). Rationale and methodology of reprogramming for generation of induced pluripotent stem cells and induced neural progenitor cells. *Int. J. Mol. Sci.* 17:594. 10.3390/ijms17040594 27104529 PMC4849048

[B100] Todd L. Hooper M. J. Haugan A. K. Finkbeiner C. Jorstad N. Radulovich N. et al. (2021). Efficient stimulation of retinal regeneration from Müller glia in adult mice using combinations of proneural bHLH transcription factors. *Cell Rep.* 37:109857. 10.1016/j.celrep.2021.109857 34686336 PMC8691131

[B101] Todd L. Jenkins W. Finkbeiner C. Hooper M. J. Donaldson P. C. Pavlou M. et al. (2022). Reprogramming Müller glia to regenerate ganglion-like cells in adult mouse retina with developmental transcription factors. *Sci. Adv.* 8:eabq7219. 10.1126/sciadv.abq7219 36417510 PMC9683702

[B102] Traxler L. Lagerwall J. Eichhorner S. Stefanoni D. D’Alessandro A. Mertens J. (2021). Metabolism navigates neural cell fate in development, aging and neurodegeneration. *Dis. Model Mech.* 14:dmm048993. 10.1242/dmm.048993 34345916 PMC8353098

[B103] Treutlein B. Lee Q. Y. Camp J. G. Mall M. Koh W. Shariati S. A. et al. (2016). Dissecting direct reprogramming from fibroblast to neuron using single-cell RNA-seq. *Nature* 534 391–395. 10.1038/nature18323 27281220 PMC4928860

[B104] Tyssowski K. M. Gray J. M. (2019). Blue light increases neuronal activity-regulated gene expression in the absence of optogenetic proteins. *eNeuro* 6:ENEURO.0085-19.2019. 10.1523/eneuro.0085-19.2019 31444226 PMC6751372

[B105] van Dyck C. H. Swanson C. J. Aisen P. Bateman R. J. Chen C. Gee M. et al. (2023). Lecanemab in Early Alzheimer’s Disease. *N. Engl. J. Med.* 388 9–21. 10.1056/NEJMoa2212948 36449413

[B106] van Lengerich B. Zhan L. Xia D. Chan D. Joy D. Park J. I. et al. (2023). A TREM2-activating antibody with a blood-brain barrier transport vehicle enhances microglial metabolism in Alzheimer’s disease models. *Nat. Neurosci.* 26 416–429. 10.1038/s41593-022-01240-0 36635496 PMC9991924

[B107] Victor M. B. Richner M. Hermanstyne T. O. Ransdell J. L. Sobieski C. Deng P. Y. et al. (2014). Generation of human striatal neurons by microRNA-dependent direct conversion of fibroblasts. *Neuron* 84 311–323. 10.1016/j.neuron.2014.10.016 25374357 PMC4223654

[B108] Victor M. B. Richner M. Olsen H. E. Lee S. W. Monteys A. M. Ma C. et al. (2018). Striatal neurons directly converted from Huntington’s disease patient fibroblasts recapitulate age-associated disease phenotypes. *Nat. Neurosci.* 21 341–352. 10.1038/s41593-018-0075-7 29403030 PMC5857213

[B109] Vierbuchen T. Ostermeier A. Pang Z. P. Kokubu Y. Südhof T. C. Wernig M. (2010). Direct conversion of fibroblasts to functional neurons by defined factors. *Nature* 463 1035–1041. 10.1038/nature08797 20107439 PMC2829121

[B110] Voitiuk K. Seiler S. T. de Melo M. P. Geng J. van der Molen T. Hernandez S. et al. (2025). A feedback-driven brain organoid platform enables automated maintenance and high-resolution neural activity monitoring. *Internet Things* 33:101671. 10.1016/j.iot.2025.101671 41522195 PMC12781996

[B111] Walie B. E. Hagemeyer C. E. Xu R. Niego B. (2026). Thrombolytic drugs for ischemic stroke: Historical perspective, state of play, and future developments. *J. Thromb Haemost.* 24 26–46. 10.1016/j.jtha.2025.09.021 41077133

[B112] Wan J. Goldman D. (2016). Retina regeneration in zebrafish. *Curr. Opin. Genet. Dev.* 40 41–47. 10.1016/j.gde.2016.05.009 27281280 PMC5135611

[B113] Wang G. Zhang D. Qin L. Liu Q. Tang W. Liu M. et al. (2024). Forskolin-driven conversion of human somatic cells into induced neurons through regulation of the cAMP-CREB1-JNK signaling. *Theranostics* 14 1701–1719. 10.7150/thno.92700 38389831 PMC10879881

[B114] Wang H. Liu S. Sun Y. Chen C. Hu Z. Li Q. et al. (2024). Target modulation of glycolytic pathways as a new strategy for the treatment of neuroinflammatory diseases. *Ageing Res. Rev.* 101:102472. 10.1016/j.arr.2024.102472 39233146

[B115] Wang H. Yang Y. Liu J. Qian L. (2021). Direct cell reprogramming: Approaches, mechanisms and progress. *Nat Rev Mol Cell Biol.* 22 410–424. 10.1038/s41580-021-00335-z 33619373 PMC8161510

[B116] Wang H. Zhao P. Zhang Y. Chen Z. Bao H. Qian W. et al. (2023). NeuroD4 converts glioblastoma cells into neuron-like cells through the SLC7A11-GSH-GPX4 antioxidant axis. *Cell Death Discov.* 9:297. 10.1038/s41420-023-01595-8 37582760 PMC10427652

[B117] Wang K. Pan S. Zhao P. Liu L. Chen Z. Bao H. et al. (2022). PTBP1 knockdown promotes neural differentiation of glioblastoma cells through UNC5B receptor. *Theranostics* 12 3847–3861. 10.7150/thno.71100 35664063 PMC9131277

[B118] Wang L. L. Zhang C. L. (2022). In vivo glia-to-neuron conversion: Pitfalls and solutions. *Dev. Neurobiol.* 82 367–374. 10.1002/dneu.22880 35535734 PMC9337910

[B119] Wang L. L. Serrano C. Zhong X. Ma S. Zou Y. Zhang C. L. (2021). Revisiting astrocyte to neuron conversion with lineage tracing in vivo. *Cell* 184 5465–81.e16. 10.1016/j.cell.2021.09.005 34582787 PMC8526404

[B120] Wang Q. Lu M. Zhu X. Gu X. Zhang T. Xia C. et al. (2022). The role of microglia immunometabolism in neurodegeneration: Focus on molecular determinants and metabolic intermediates of metabolic reprogramming. *Biomed. Pharmacother.* 153:113412. 10.1016/j.biopha.2022.113412 36076537

[B121] Wang W. Liu X. Wang D. C. (2024). Single-cell and spatial alterations of neural cells and circuits in clinical and translational medicine. *Clin. Transl. Med.* 14:e1696. 10.1002/ctm2.1696 38812092 PMC11136700

[B122] Wapinski O. L. Lee Q. Y. Chen A. C. Li R. Corces M. R. Ang C. E. et al. (2017). Rapid chromatin switch in the direct reprogramming of fibroblasts to neurons. *Cell Rep.* 20 3236–3247. 10.1016/j.celrep.2017.09.011 28954238 PMC5646379

[B123] Wapinski O. L. Vierbuchen T. Qu K. Lee Q. Y. Chanda S. Fuentes D. R. et al. (2013). Hierarchical mechanisms for direct reprogramming of fibroblasts to neurons. *Cell* 155 621–635. 10.1016/j.cell.2013.09.028 24243019 PMC3871197

[B124] Wu Z. Parry M. Hou X. Y. Liu M. H. Wang H. Cain R. et al. (2020). Gene therapy conversion of striatal astrocytes into GABAergic neurons in mouse models of Huntington’s disease. *Nat. Commun.* 11:1105. 10.1038/s41467-020-14855-3 32107381 PMC7046613

[B125] Wytock T. P. Motter A. E. (2024). Cell reprogramming design by transfer learning of functional transcriptional networks. *Proc. Natl. Acad. Sci. U. S. A.* 121:e2312942121. 10.1073/pnas.2312942121 38437548 PMC10945810

[B126] Xiao D. Jin K. Qiu S. Lei Q. Huang W. Chen H. et al. (2021). In vivo regeneration of ganglion cells for vision restoration in mammalian retinas. *Front. Cell Dev. Biol.* 9:755544. 10.3389/fcell.2021.755544 34671605 PMC8520940

[B127] Xu H. Lin S. Zhou Z. Li D. Zhang X. Yu M. et al. (2023). New genetic and epigenetic insights into the chemokine system: The latest discoveries aiding progression toward precision medicine. *Cell Mol. Immunol.* 20 739–776. 10.1038/s41423-023-01032-x 37198402 PMC10189238

[B128] Xu J. Fang S. Deng S. Li H. Lin X. Huang Y. et al. (2023). Generation of neural organoids for spinal-cord regeneration via the direct reprogramming of human astrocytes. *Nat. Biomed. Eng.* 7 253–269. 10.1038/s41551-022-00963-6 36424465 PMC12889199

[B129] Xue Y. Ouyang K. Huang J. Zhou Y. Ouyang H. Li H. et al. (2013). Direct conversion of fibroblasts to neurons by reprogramming PTB-regulated microRNA circuits. *Cell* 152 82–96. 10.1016/j.cell.2012.11.045 23313552 PMC3552026

[B130] Xue Y. Qian H. Hu J. Zhou B. Zhou Y. Hu X. et al. (2016). Sequential regulatory loops as key gatekeepers for neuronal reprogramming in human cells. *Nat. Neurosci.* 19 807–815. 10.1038/nn.4297 27110916 PMC4882254

[B131] Yang Y. Cui J. Kong Y. Hou Y. Ma C. (2024). Organoids: New frontiers in tumor immune microenvironment research. *Front. Immunol.* 15:1422031. 10.3389/fimmu.2024.1422031 39136020 PMC11317300

[B132] Yao K. Qiu S. Wang Y. V. Park S. J. H. Mohns E. J. Mehta B. et al. (2018). Restoration of vision after de novo genesis of rod photoreceptors in mammalian retinas. *Nature* 560 484–488. 10.1038/s41586-018-0425-3 30111842 PMC6107416

[B133] Yue F. Feng S. Lu C. Zhang T. Tao G. Liu J. et al. (2021). Synthetic amyloid-β oligomers drive early pathological progression of Alzheimer’s disease in nonhuman primates. *iScience* 24:103207. 10.1016/j.isci.2021.103207 34704001 PMC8524197

[B134] Zhang H. Guo Y. Yang Y. Wang Y. Zhang Y. Zhuang J. et al. (2023). MAP4Ks inhibition promotes retinal neuron regeneration from Müller glia in adult mice. *NPJ Regen. Med.* 8:36. 10.1038/s41536-023-00310-6 37443319 PMC10344969

[B135] Zhang Q. J. Li J. J. Lin X. Lu Y. Q. Guo X. X. Dong E. L. et al. (2017). Modeling the phenotype of spinal muscular atrophy by the direct conversion of human fibroblasts to motor neurons. *Oncotarget* 8 10945–10953. 10.18632/oncotarget.14641 28099929 PMC5355236

[B136] Zhang T. Ke W. Zhou X. Qian Y. Feng S. Wang R. et al. (2019). Human neural stem cells reinforce hippocampal synaptic network and rescue cognitive deficits in a mouse model of Alzheimer’s disease. *Stem Cell Rep.* 13 1022–1037. 10.1016/j.stemcr.2019.10.012 31761676 PMC6915849

[B137] Zhao J. He H. Zhou K. Ren Y. Shi Z. Wu Z. et al. (2012). Neuronal transcription factors induce conversion of human glioma cells to neurons and inhibit tumorigenesis. *PLoS One* 7:e41506. 10.1371/journal.pone.0041506 22859994 PMC3409237

[B138] Zhou H. Su J. Hu X. Zhou C. Li H. Chen Z. et al. (2020). Glia-to-Neuron conversion by CRISPR-CasRx alleviates symptoms of neurological disease in mice. *Cell* 181 590–603.e16. 10.1016/j.cell.2020.03.024 32272060

[B139] Zhou M. Tao X. Sui M. Cui M. Liu D. Wang B. et al. (2021). Reprogramming astrocytes to motor neurons by activation of endogenous Ngn2 and Isl1. *Stem Cell Rep.* 16 1777–1791. 10.1016/j.stemcr.2021.05.020 34171285 PMC8282467

[B140] Zhu Y. Huang R. Wu Z. Song S. Cheng L. Zhu R. (2021). Deep learning-based predictive identification of neural stem cell differentiation. *Nat. Commun.* 12:2614. 10.1038/s41467-021-22758-0 33972525 PMC8110743

[B141] Zhu Y. Tchkonia T. Pirtskhalava T. Gower A. C. Ding H. Giorgadze N. et al. (2015). The Achilles’ heel of senescent cells: From transcriptome to senolytic drugs. *Aging Cell* 14 644–658. 10.1111/acel.12344 25754370 PMC4531078

